# A Competition‐Based Strategy for the Isolation of an Anti‐Idiotypic Blocking Module and Fine‐Tuning for Conditional Activation of a Therapeutic Antibody

**DOI:** 10.1002/biot.202400432

**Published:** 2024-12-10

**Authors:** Jan Habermann, Dominic Happel, Adrian Bloch, Charles Shin, Harald Kolmar

**Affiliations:** ^1^ Institute for Organic Chemistry and Biochemistry Technical University of Darmstadt Darmstadt Hesse Germany; ^2^ Department of Biomedical Engineering Johns Hopkins University Baltimore Maryland USA; ^3^ Centre for Synthetic Biology Technical University of Darmstadt Darmstadt Hesse Germany

**Keywords:** antibody engineering, antibody masking, conditional antibody activation, tumor protease

## Abstract

The masking of therapeutic antibodies by the installation of a blocking module that can be removed under certain physiological conditions, is becoming increasingly important to improve their safety and toxicity profile. To gain access to such masking units, we used chicken immunization in combination with yeast surface display and a competition‐based FACS screening campaign to obtain anti‐idiotypic single‐chain Fv (scFv) fragments. This approach promotes the identification of functional masking units, since specificity and high affinity do not necessarily guarantee a paratope blocking effect. This strategy was used to isolate a scFv masking unit for the therapeutic antibody 6G11 (BI‐1206), which is currently in clinical trials for the treatment of B‐cell lymphoma to block the inhibitory Fcγ receptor IIB (CD32b). N‐terminal fusion of the anti‐idiotypic scFv to the 6G11 light chain successfully abolished binding to FcγRIIB in vitro. For conditional activation, a cleavable linker for the tumor‐associated protease MMP‐9 was implemented. To improve demasking efficiency, the affinity of the scFv mask was attenuated through rational design. The substitution of one key amino acid in the masking scFv reduced the affinity toward the 6G11 paratope by factor 10 but still mediated 9800‐fold blocking of receptor binding. Proteolytic demasking allowed full recovery of therapeutic antibody function in vitro, supporting the concept of conditional antibody activation using this anti‐idiotypic binding module.

## Graphical Abstract and Lay Summary

A recent approach in restricting antibody binding to the tumor‐site is the implementation of masking domains in combination with protease cleavable linkers. We report a strategy for the isolation of scFv‐based affinity masking domains using chicken immunization and a competitive FACS screening.

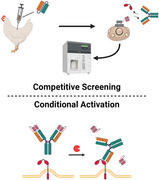

**Abbreviations**
BLI: biolayer interferometry
CDR: complementary determining region
ELISA: enzyme‐linked immunosorbent assay
FACS: fluorescence activated cell sorting
FcγRIIB/CD32b: inhibitory Fc gamma receptor IIB
FR: framework region
mAb: monoclonal antibody
MMP: matrix metalloproteinase
PBS: phosphate buffered saline
RT: room temperature
scFv: single‐chain fragment variable
SEC: size exclusion chromatography
TBS: Tris buffered saline
tGFP: turbo green fluorescent protein
TME: tumor microenvironment
YSD: yeast surface display

## 1 Introduction

In recent years, multiple strategies have been reported to generate next‐generation therapeutic antibodies, particularly for cancer treatment. One of the major efforts is to direct the antibody to tumor tissue while sparing healthy cells. Among other options, this can be achieved by conditional activation of a functionally masked therapeutic antibody [1]. To this end, a masking domain is fused via a flexible linker to the amino terminus of the antibody heavy or light chain in close proximity to the antigen binding site thereby preventing the antibody from binding in healthy tissue. Upon entering the tumor microenvironment (TME), the linker is cleaved by a tumor‐specific protease, and antibody binding is restored. Masking domains can be categorized into two different types: affinity‐based and steric hindrance‐based. The latter does not rely on an interaction with the paratope of the antibody to block target binding. Therefore, the masking domain can be easily and ubiquitously transferred to different monoclonal antibodies (mAbs) [1]. However, their masking efficiency can vary based on the antibody, and implementation of individual adjustments can be tedious. In contrast, affinity‐based masking domains interact specifically with the paratope region of the antibody, hence termed anti‐idiotypic. They are tailor‐made for each antibody and the masking efficiency can be adjusted to the needs of the application by modulating the affinity of the masking domain [2]. Several affinity peptide masks and strategies exist, for example, utilizing one peptide per paratope like in the anti‐EGFR probody PB1 by CytomX [3] or even a bivalent peptide‐DNA conjugate [4]. Additionally, scFvs and nanobodies have been explored as affinity‐based anti‐idiotype masking domains [5, 6, 7].

Eventually, conditional activation of masked antibodies can then be achieved by rendering the masking unit pH dependent [8] or by exploiting TME associated proteases for demasking that cleave the masking unit off resulting in regain of antigen binding by the therapeutic antibody. To this end, matrix metalloproteases (MMPs) have frequently been reported to act as activators of masked antibodies [3, 4, 5]. MMP‐9 is the most studied member and plays important roles in early embryonal development as well as immune cell recruitment in the lung and wound healing. Dysregulation of MMP‐9 expression is also associated with different diseases such as cardiovascular diseases, arthritis, and diabetes [9]. In different cancer types, increased MMP levels have been correlated with worse overall survival [10, 11, 12, 13]. To utilize the difference in protease expression in the TME, different protease cleavable linkers have been developed to attach masking domains to mAbs [3, 5].

Notably, by generating anti‐idiotype masking units that specifically and potently block the antigen binding site and linking them to a therapeutic antibody, significant improvements in tumor selectivity can be achieved [1, 5]. We reasoned that a generic strategy might be useful to gain reliable and rapid access to these types of masking units and therefore aimed to develop a screening strategy to obtain masking units from the antibody repertoire of a chicken immunized with the therapeutic antibody for which a masking module is sought.

As an experimental example, we have chosen a therapeutic antibody that is relevant to a tumor escape strategy in B‐cell lymphoma. Upon the treatment of B‐cell lymphoma with the commonly used anti‐CD20 antibody rituximab, internalization from the surface of B cells was observed, where the rate of internalization directly correlated with the expression of the inhibitory Fc gamma receptor FcγRIIB (CD32b) in these tumor cells [14, 15]. As a consequence, surface‐bound rituximab is internalized upon cis binding to FcγRIIB with its Fc part (Figure S1). Not only is the CD20:rituximab complex removed from the cell surface and degraded in the lysosome, but FcγRIIB‐bound rituximab is also unable to interact in trans with activating Fc receptors on effector cells that mediate tumor cell killing [16]. The inhibitory FcγR plays an important role in regulating activation thresholds [17]. In B cells, FcγRIIB is a major checkpoint during development, survival, and antibody production [18, 19], whereas dysregulation can lead to autoimmune diseases. Hence, FcγRIIB is a complex but promising target not only for tumor therapy but also for the treatment of autoimmune diseases like rheumatoid arthritis which could benefit from agonistic engagement and thus inhibitory signaling in autoreactive B cells [20, 21, 22].

6G11 (BI‐1206) is a fully human antagonistic therapeutic antibody directed toward FcγRIIB with no cross‐reactivity to FcγRIIA, other human tissues, or to FcγRIIB of other species [23]. 6G11 not only inhibits ITIM signaling on effector cells but also prevents the internalization of co‐administered therapeutic antibodies in B‐cell lymphoma such as rituximab, which forces rituximab‐resistant tumors to regain responsiveness upon co‐administration with 6G11 in vitro and in preclinical in vivo studies [24]. The aim of three ongoing clinical phase 1/2a studies is the investigation of side effects and clinical benefits in combination with rituximab (anti‐CD20), pembrolizumab (anti‐PD‐1), or trastuzumab (anti‐HER2) (ClinicalTrials.gov: NCT03571568; NCT04219254, NCT05555251). As an intermediate result for the non‐Hodgkin's lymphoma study, IV administered 6G11 (30–100 mg) in combination with rituximab showed positive responses in patients with no immediate severe side effects so far [24]. As described above, however, FcγRIIB is involved in many processes and a thorough assessment of the 6G11 safety profile still requires finalization and analysis of the current studies. Possible side effects could be circumvented by only localized 6G11‐engagement and conditional activation in the tumor microenvironment. As FcγRIIB is expressed on all circulating CD20+ B lymphocytes, not just lymphoma cells, restricting the function of the FcγRIIB mAb to the TME via conditional blocking/activation may help to reduce off‐target and sink effects.

Besides overexpression of FcγRIIB on target cells and effector cells in the TME [25, 26], increased levels of MMPs are also reported for B‐cell lymphoma [27, 28, 29, 30, 31].

In this study, we report the isolation of anti‐idiotype chicken‐derived scFvs directed against 6G11 from an immune library utilizing yeast surface display (YSD) and competitive fluorescence activated cell sorting (FACS). Two classes of scFvs were isolated that either interfere with the 6G11:FcγRIIB interaction or bind to 6G11 regardless of FcγRIIB presence. We fused an interaction‐blocking scFv to the light chain of 6G11 utilizing an MMP‐9 cleavable linker and analyzed masking as well as demasking efficiency. To fully recover antibody function, we performed affinity attenuation of the masking unit by structure‐guided identification of key amino acids in the interface for selective amino acid substitutions. This allowed conditional activation of the 6G11 antibody by MMP‐9 in vitro.

## 2 Results

### 2.1 Immunization and Yeast Library Generation

**FIGURE 1:**
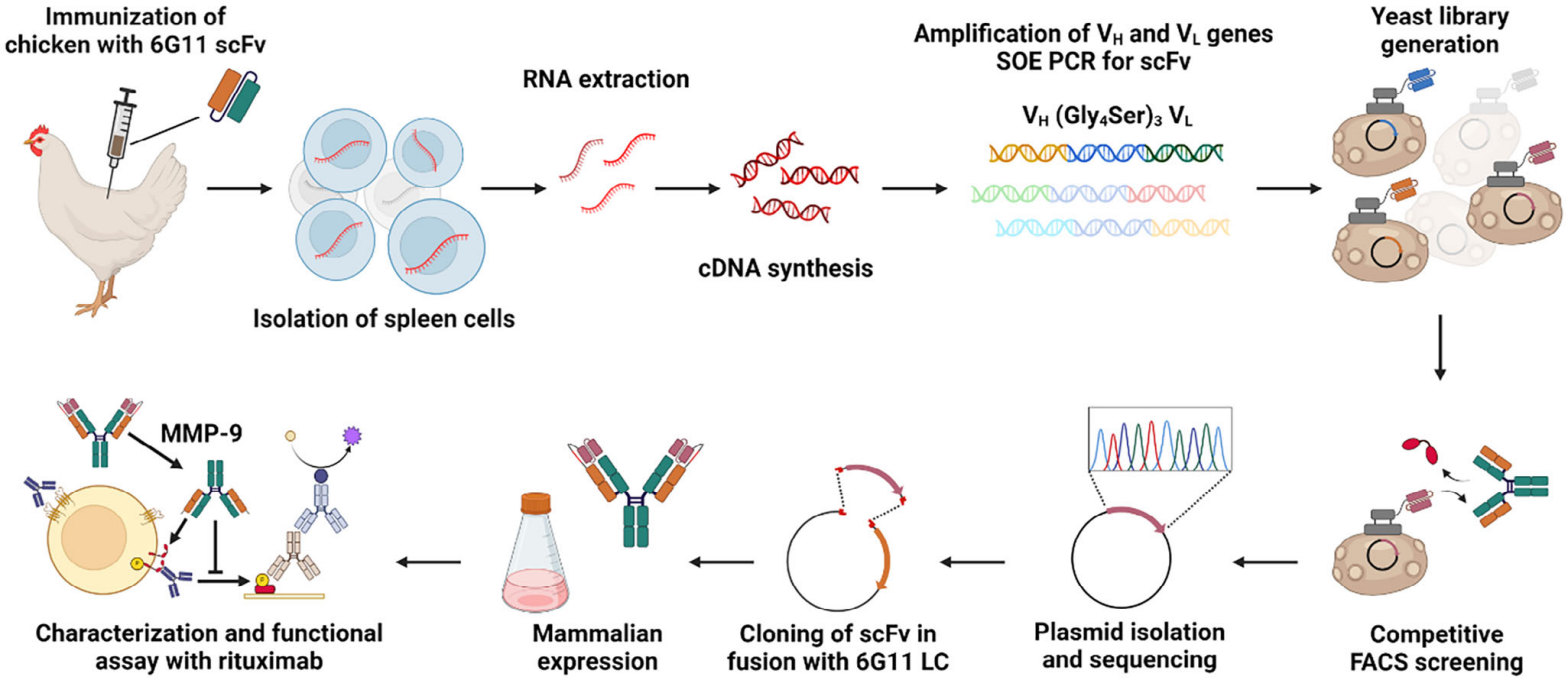
Workflow to generate and test a scFv masked 6G11. Chicken immunization was performed with 6G11 scFv. cDNA was synthesized from isolated RNA and used for amplification of the chicken V_H_ and V_L_ repertoire. scFv genes were generated by overlap extension PCR and integrated in a yeast surface display plasmid via homologous recombination. In a competitive fluorescence activated cell sorting (FACS) campaign, a scFv blocking the binding of FcγRIIB to 6G11 was isolated, reformatted as N‐terminal light chain fusion to 6G11, and purified from Expi293F HEK cells via Protein A chromatography. Subsequently, the fusion protein was characterized and tested by in vitro experiments. Created with BioRender.

To obtain anti‐idiotype antibodies rather than antibodies directed against the constant domains of 6G11, a scFv variant was generated based on the 6G11 V_H_ and V_L_ domain sequences listed in the patent [32], connected by an (G_4_S)_3_ linker (Figure 1). Transformation of *Escherichia coli* with the pET30 derived plasmid resulted in expression of a 6G11 scFv with N‐terminal His‐tag and C‐terminal Strep‐tag. The scFv was subsequently purified via IMAC and StrepTactin affinity chromatography. Immunization of a chicken with the 6G11 scFv was carried out according to standard protocols and resulted in a high antibody titer after the 4th booster immunization (Figure S2). After RNA isolation from chicken spleen cells and cDNA synthesis, V_H_ and V_L_ sequences were amplified and fused together via PCR thereby introducing a (G_4_S)_3_ linker. The resulting PCR product was utilized in combination with a yeast surface display plasmid containing Aga2p, HA tag, c‐myc tag, and in addition a T2A and tGFP sequence [33]. The intracellular tGFP expression was utilized during the screening process as a marker for scFv surface expression (Figure S3). Upon transformation into *S. cerevisiae* EBY100, 1.3•10^9^ individual clones were obtained.

### 2.2 Library Sorting and Characterization of Binders

**FIGURE 2:**
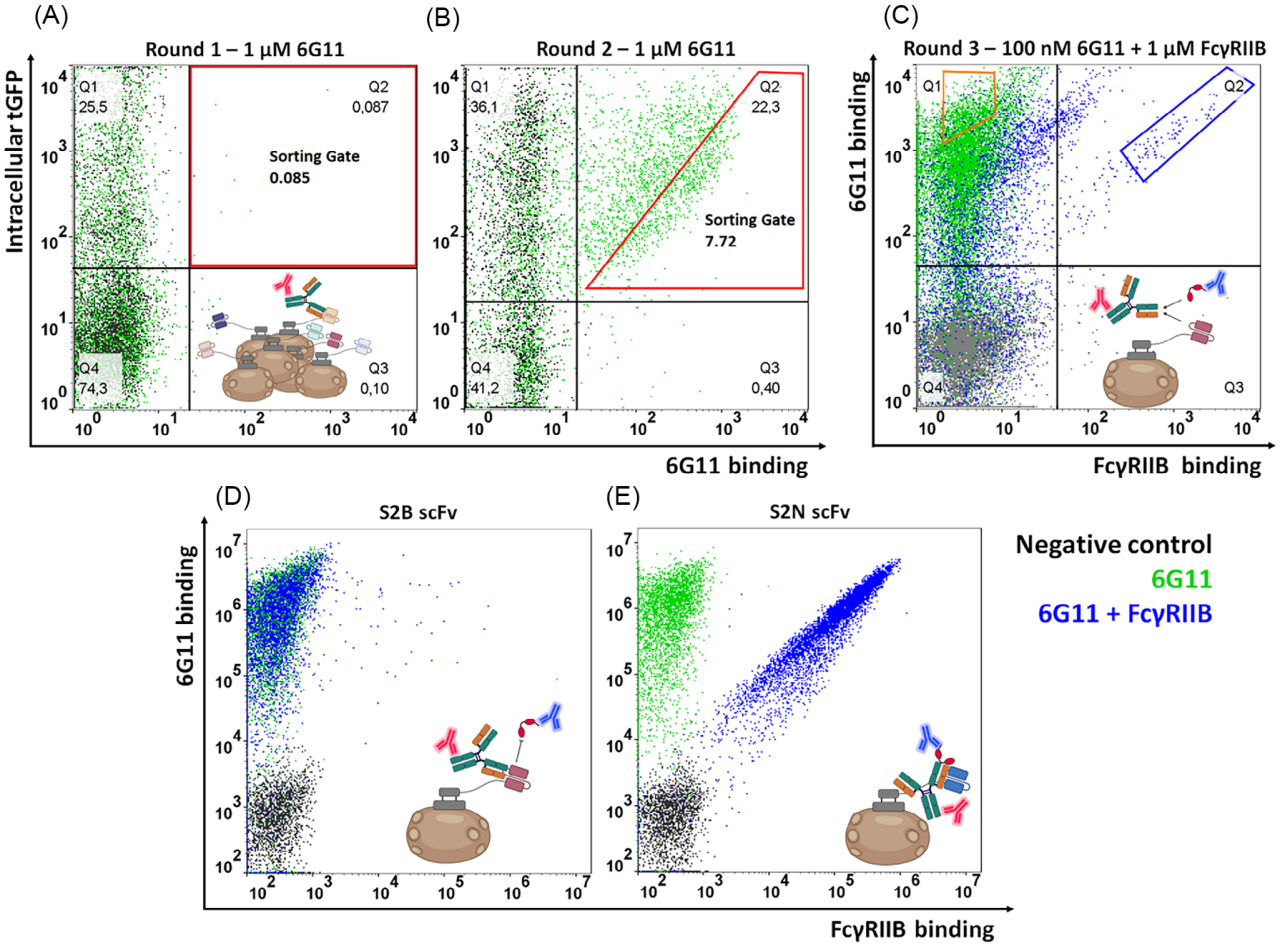
Flow cytometry dot plots of the chicken scFv immune library screened toward 6G11 binding. (A and B) Screening rounds 1 and 2 toward 6G11 binding. The cells depicted in the dot plots are gated on viable and single cells. Chicken scFv surface presentation is depicted on the y‐axis indicated by intracellular tGFP expression, while 6G11 antigen binding (anti‐human Fc‐PE) is shown on the x‐axis. The negative control (anti‐human Fc‐PE) is depicted in grey, whereas cells incubated with 1 µM 6G11 are shown in green. The cells sorted are displayed in the red sorting gate. (C) Round 3 of screening toward blocking and non‐blocking anti‐6G11 chicken scFvs. 6G11 binding (anti‐human Fc‐PE) is shown on the y‐axis, while FcγRIIB binding (anti‐his Alexa Fluor647) is depicted on the x‐axis. The negative control (anti‐human Fc‐PE and anti‐his Alexa Fluor647) is shown in gray, while cells incubated with 100 nM 6G11 are depicted in green and cells co‐incubated with 100 nM 6G11 and 1 µM FcγRIIB are shown in blue. The sorting gate for the isolation of scFvs binding only to 6G11 and the gate for variants revealing signals for 6G11 and FcγRIIB are shown in orange and blue, respectively. (D and E) Single clone analysis of blocking anti‐6G11 scFv clone 2 (S2B) and non‐blocking anti‐6G11 scFv clone 2 (S2N). Staining was performed equivalent to round 3.

The library was sorted two rounds for binding toward full‐length 6G11 [32] (1 µM used for staining) containing the PGLALA Fc mutations to prevent the interaction with FcγRIIB during the subsequent screening process (Figure 2A,B). After the enrichment of a binding population, 6G11 (100 nM) was premixed with a 10‐fold molar excess of FcγRIIB and added to the outcome of the second sorting round (Figure 2C). The gating strategy was adapted to ensure only surface‐presenting cells were included in the binding analysis. One population in this sample revealed strong binding toward 6G11, comparable to the control only incubated with 6G11, while cells with binding toward the 6G11:FcγRIIB complex were also observed (Figure 2C). scFvs binding only to 6G11 were isolated in the orange gate, while variants displaying a binding signal for the 6G11:FcγRIIB complex were isolated in the blue gate. Single clones from both screening approaches were investigated for the capacity to bind 6G11 in the presence of FcγRIIB (Figure 2D,E). Among others, clone S2B, isolated from the population with 6G11 but no FcγRIIB signal, prevented additional binding of FcγRIIB to 6G11 (Figure 2D), indicating that the scFv competes with the receptor for 6G11 binding. S2N instead, isolated from the approach recognizing the 6G11:FcγRIIB complex, did not prevent a FcγRIIB signal (Figure 2E). Not unexpectedly, both scFvs displayed different CDR sequences in their V_H_ and V_L_ domains (Figure S4). Both scFvs were reformatted via golden gate cloning in a pET30 expression plasmid, thereby introducing a N‐terminal His‐tag and a C‐terminal Strep‐tag. Afterward, both scFvs were solitarily expressed in *E. coli* and purified via IMAC and StrepTactin chromatography. The binding interaction between the scFvs and 6G11 was investigated via ELISA and revealed an EC_50_ of 28.7 nM for S2B and 3.9 nM for S2N (Figure S5A, Table S1). Their melting points were determined to be 59.0°C and 51.5°C, respectively (Figure S11A, Table S1). Furthermore, both scFvs were analyzed for 6G11 binding specificity. Biolayer interferometry (BLI) was used to test the binding of the two scFvs to three unrelated antibodies (Figure S6). scFv specificity was tested in the presence of kappa (matuzumab [34]) and lambda (hCP‐LCE [35]) light chains as well as rituximab [36], to exclude undesired effects during functional assays. Each control antibody was loaded to a Protein A biosensor and subsequently incubated with 2 µM of each scFv. While binding toward 6G11 is evident for both scFvs, no engagement of the different controls was observed. In addition, epitope binning was performed since both scFvs revealed vastly different binding behaviors regarding binding the 6G11:FcγRIIB complex (Figure S7). For this, 6G11 was immobilized on a Protein A biosensor tip which was sequentially incubated with both scFvs. After the association of the S2B scFv, a significant increase in binding was observed upon incubation with S2N, corroborating the notion that both scFvs recognize different epitopes on 6G11.

### 2.3 Construction and Characterization of 6G11 Light Chain Fusions

Both scFvs were fused to the N‐terminus of the light chain via an MMP‐9 cleavable linker [5], from here on, these light chain fusions are referred to as S2B‐6G11 and S2N‐6G11. After expression in mammalian cells and Protein A purification, analysis via SDS‐PAGE revealed expected chain sizes (Figure S12A–C). Next, linker stability in presence of unrelated but disease‐related proteases uPA and matriptase was investigated (Figure S12B,C). Only incubation with MMP‐9 resulted in the cleavage of both light chain fusions and showed the expected band pattern. As expected, unmodified 6G11 was not proteolytically digested (Figure S12A). Further biophysical characterization via thermal shift assay (Figure S11C,D) revealed a melting point reduction by 1°C and 3°C for S2B‐6G11 and S2N‐6G11, respectively, compared to 6G11 (56.5°C). Upon MMP‐9 cleavage, the melting point of both variants increased to the level of 6G11. Analysis of aggregation behavior via analytical size exclusion chromatography (SEC) revealed a similar amount of aggregates for 6G11 and S2B‐6G11 post and prior MMP‐9 digestion (Figure S10A), while the aggregation levels for S2N‐6G11 were increased regardless of the linker state (Figure S10B).

To investigate the masking effect of both scFvs in the light chain fusions, affinities were determined via BLI (Figure S9B–E). Binding analysis revealed a K_D_ of 32.1 nM for 6G11 (Figure S9A), which is comparable to the previously published data [23]. In contrast, the K_D_ of S2N‐6G11 is 8.3 nM with a slower dissociation compared to 6G11 (Figure S9B). In case of S2B‐6G11, no binding was observed before and after MMP‐9 cleavage, most likely due to the fact that the scFv was retained on the 6G11 paratope after cleavage (Figure S9C,D). To demonstrate that binding of S2B‐6G11 can be re‐established after MMP‐9 digestion, a 6G11‐based affinity purification column was generated. After applying the digestion mixture to the column, the removal of approximately 50% of the scFv was achieved (Figure S12D), and a K_D_ for unmasked 6G11 of 5.9 nM was calculated (Figure S9E).

**FIGURE 3:**
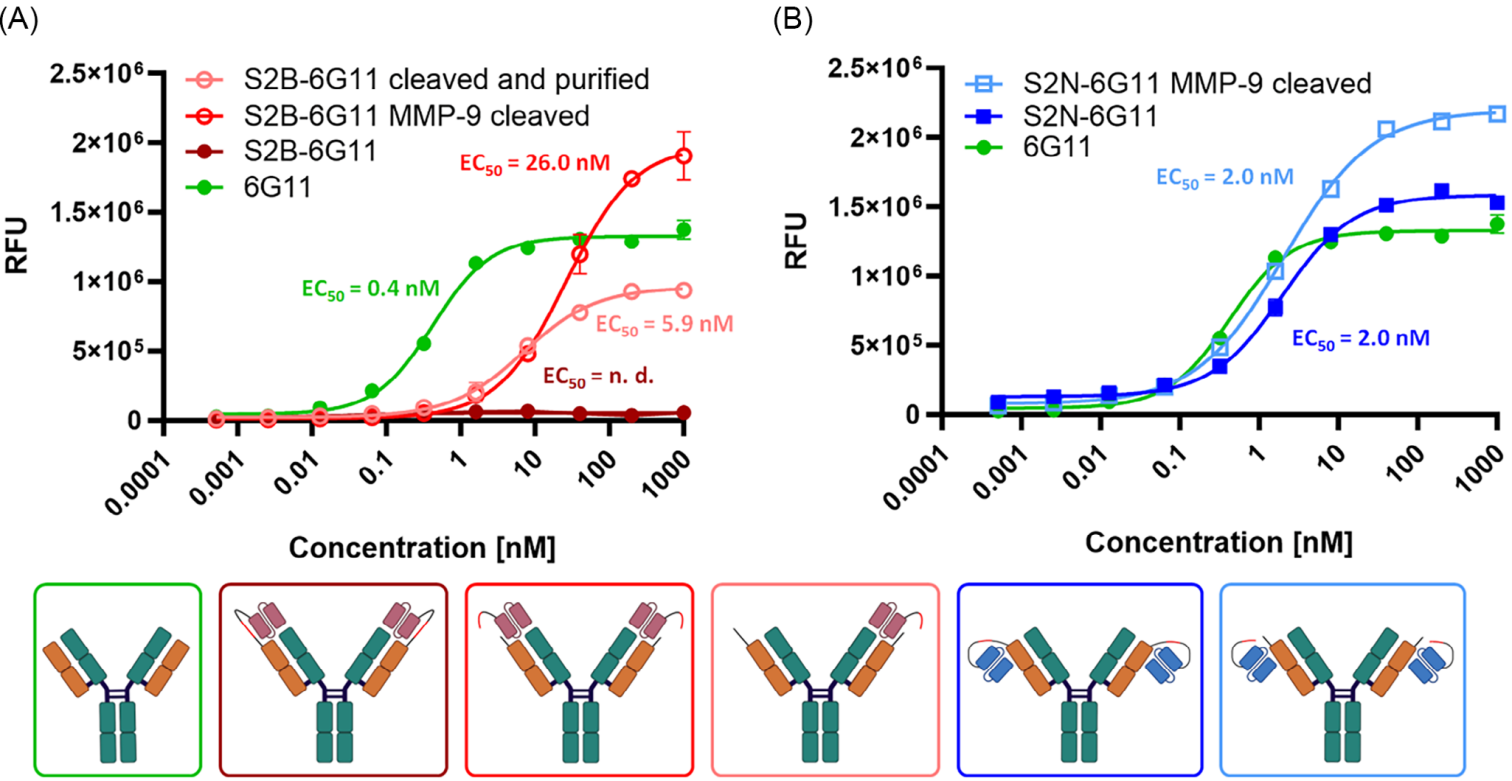
EC_50_ determination on Raji cells of anti‐6G11 chicken scFv fused to the light chain of 6G11 via an MMP‐9 cleavable linker. (A) Binding curves of S2B light chain fusion constructs to Raji cells. The positive control is represented by unmodified 6G11, shown in green. The binding curve of S2B fused to the light chain of 6G11 via the MMP‐9 cleavable linker is shown in dark red. Binding after cleavage with MMP‐9 is depicted in red, while the binding curve after removal of the scFv is depicted in light red. (B) Binding curves of S2N light chain fusions to Raji cells. The positive control is shown in green. The blue curve represents S2N fused by the MMP‐9 cleavable linker to the 6G11 light chain. Binding after MMP‐9 cleavage is shown in light blue.

To further analyze the blocking and demasking efficiencies of both light chain fusions, cell binding experiments on FcγRIIB expressing Raji cells were performed (Figure 3). For 6G11 on Raji cells, an EC_50_ of 0.4 nM was determined, which is in line with the previously published data [23]. S2N‐6G11 prior and post MMP‐9 digestion displayed an EC_50_ of 2 nM (Figure 3B). While the curve profile prior to MMP‐9 digestion was comparable to 6G11, higher RFU signals for concentrations above 1 nM were observed after MMP‐9 digestion. In contrast, an EC_50_ for S2B‐6G11 before MMP‐9 digestion could not be calculated, since binding even at the highest concentration of 1 µM was not detectable (Figure 3A). The EC_50_ after MMP‐9 cleavage was determined to be 26 nM, with a higher RFU signal at higher concentrations compared to 6G11. Upon partial removal of the scFv, the EC_50_ was calculated to be 5.9 nM and the RFU signal was decreased compared to the unmodified 6G11 binding signal.

To validate that the scFv mask sufficiently blocks the 6G11 and FcγRIIB interaction in vitro, the phosphorylation state of the receptor was examined via Western blot analysis (Figure S13). Upon rituximab stimulation, phosphorylation of the receptor ITIM occurs [15]. Hence, Raji cells were co‐incubated with rituximab, 6G11, or one of the 6G11 light chain fusions prior and post MMP‐9 digestion. As previously shown by Roghanian et al. [23], FcγRIIB was not activated by rituximab in the presence of the antagonistic 6G11. Phosphorylation could be induced if 6G11 was masked by the blocking scFv S2B but not by S2N. After MMP‐9 cleavage of the mask's linker, inhibition of FcγRIIB was not significantly restored despite full proteolytic conversion (Figure S12B). Here, the cleaved‐off scFv completely abolished 6G11 function in the phosphorylation assay, most likely since it retained binding to 6G11. As described above, the cleaved‐off scFv was partially removed via a 6G11‐based affinity column (Figure S12D) to demonstrate conditional restoration of 6G11 activity. The excess of unoccupied 6G11 in the sample was then able to prevent FcγRIIB binding to the rituximab Fc.

### 2.4 Rational Design to Modulate Masking Behavior

**FIGURE 4:**
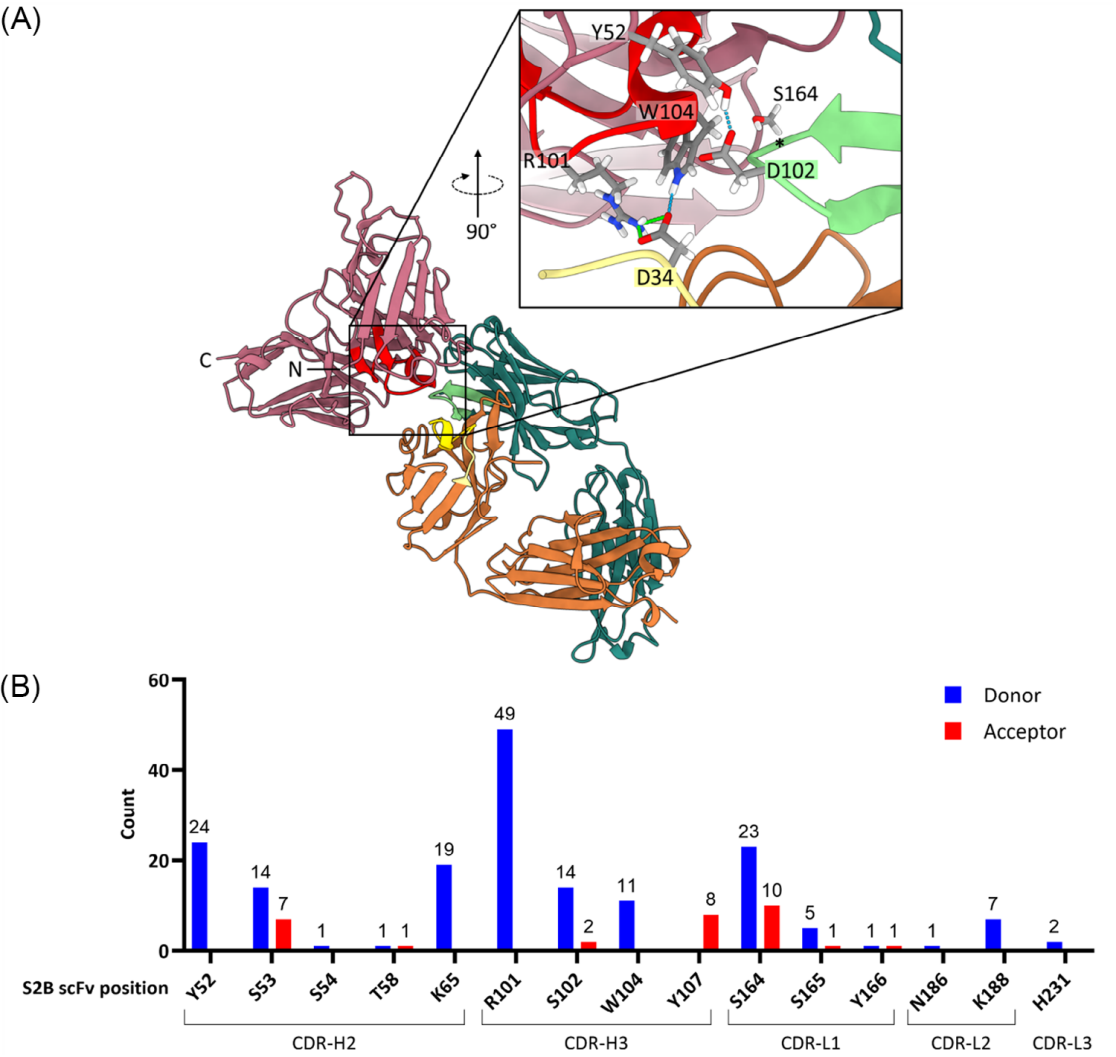
Visualization and analysis of the anti‐idiotypic S2B‐6G11 interface. (A) Representative AlphaFold 3 model of S2B scFv binding to 6G11 visualized in ChimeraX. S2B scFv is shown in mauve with CDR‐H3 highlighted in red. 6G11 V_L_ and C_L_ are shown in orange with CDR‐L1 highlighted in yellow. 6G11 V_H_ and C_H_1 are shown in dark green with CDR‐H3 highlighted in light green. Zoomed in view shows predicted salt bridges in green and hydrogen bonds in blue between scFv sidechains and the CDRs of 6G11. Position of 6G11 Y101 and hydrogen bond with S2B S164 marked by an asterisk. (B) Occurrence of hydrogen bonds and salt bridges with 6G11 summarized per scFv amino acid position from 25 AlphaFold 3 simulations.

**FIGURE 5:**
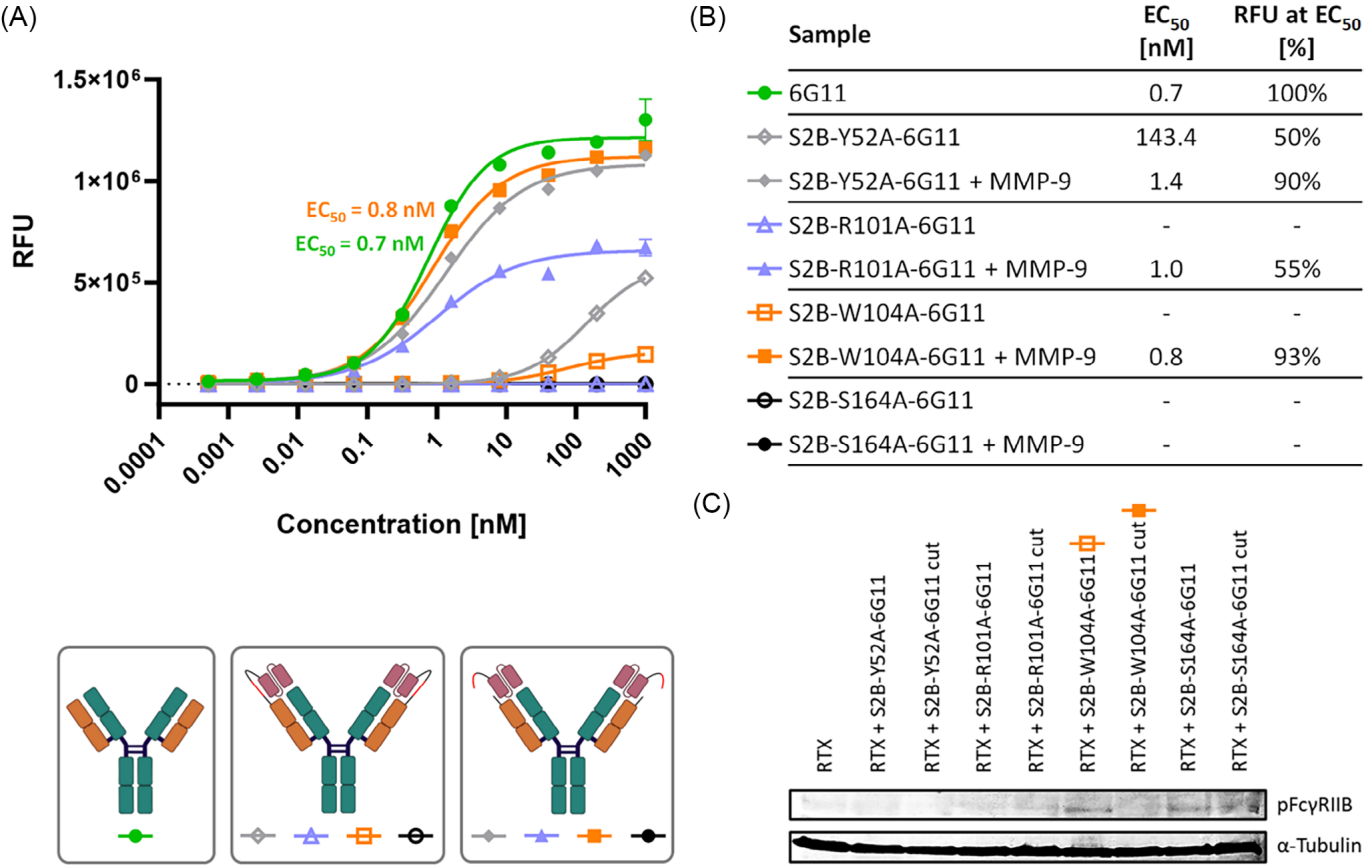
Evaluation of S2B‐6G11 mutants in terms of on‐cell EC_50_ and impact on FcγRIIB phosphorylation status in Raji cells. (A) EC_50_ determination on Raji cells of 6G11 in fusion with S2B scFv mutants. The binding curve of unmodified 6G11 is shown as a reference in green. The empty and filled symbols in matching colors mark light chain fusion constructs as prior and post MMP‐9 digestion, respectively. (B) Table summarizing the EC_50_ values and the RFU signal recovery at the EC_50_ point normalized to 6G11. (C) Western blot analysis of the FcγRIIB phosphorylation state in Raji cells in the presence of rituximab (RTX) alone and in combination with S2B‐6G11 variants prior and post MMP‐9 digestion. Staining with anti‐CD32B (phospho Y292) antibody from rabbit and anti‐rabbit‐IgG‐AP. Alpha tubulin was stained as the loading control.

To improve the restoration of 6G11 functionality without the need for artificial mask removal after MMP‐9 cleavage, affinity attenuation of the S2B scFv was performed through rational design. The scFv:6G11 interface (Figure 4A) was simulated via AlphaFold 3 [37] and the occurrence of simulated H‐bonds and salt bridges between the proteins was summarized for 25 models per scFv amino acid residue (Figure 4B). As the most prominent candidate for individual alanine substitution emerged R101 in CDR‐H3, potentially forming a salt bridge with D34 in CDR‐L1 of 6G11 (Figure 4A). Since tryptophan residues in CDRs are often revealed as hotspots for target affinity [38], W104, which could also form a hydrogen bond with D34 or engage in a hydrophobic interaction with Y51 in FR‐L2 of 6G11 (not shown), was chosen from CDR‐H3 as well. To further variegate the range of attenuation, positions in neighboring scFv CDRs, which form hydrogen bonds with sidechains in 6G11 CDR‐H3, were also selected: Y52 and S164 in CDR‐H2 and CDR‐L1, respectively. To analyze the individual impact of these four substitutions on thermal stability and 6G11 binding, the scFv mutants were solitary expressed and characterized (Table S1). Melting points at 59.5°C–64.5°C comparable to the original S2B were observed (Figure S11B). Affinity determination of the scFv mutants via BLI revealed 4‐ to 10‐fold attenuation, except for the S164A substitution in CDR‐L1 with no apparent change in affinity (Figure S8, Table S1), and similar trends were observed for ELISA (Figure S5B,C). Subsequent fusion of the S2B mutants to the light chain of 6G11 resulted in aggregation below 4% comparable to unmodified 6G11, with a tendency for monomer decrease upon MMP‐9 digestion (Figure S10C–F), while melting points (57.0°C–58.0°C) were comparable to the previous S2B construct (Figure S11E–H, Table S1). BLI measurement showed no binding of FcγRIIB to any of the four mutated scFv‐6G11 light chain fusions (Figure S9F–I). Following MMP‐9 incubation and quantitative conversion (Figure S12E), binding to S2B‐R101A‐6G11 and S2B‐S164A‐6G11 remained limited, while S2B‐Y52A‐6G11 and S2B‐W104A‐6G11 achieved significant receptor binding restoration (Figure S9J–M, Table S1). On‐cell binding analysis of the uncleaved light chain fusions showed strong to moderate signal reduction compared to the free 6G11 control, while S2B‐Y52A‐6G11 showed the weakest blocking effect above 40 nM (Figure 5A). Except for the S2B S164A mask, an increase in cell binding was measured after MMP‐9 linker cleavage, with calculated EC_50_s near 1 nM comparable to 6G11. At this concentration, cleaving off the Y52A and W104A versions of S2B resulted in 90% recovery of the binding signal relative to the free antibody, while digested S2B‐R101A‐6G11 was restored to ∼55% (Figure 5B). For the S2B‐W104A‐6G11 construct, a 9800‐fold in blocking was calculated, when comparing the concentration at the highest binding signal for the undigested construct with the concentration of the cleaved sample at equal signal strength. The blocking efficiency of the mutated S2B light chain fusions and MMP‐9 mediated recovery of the inhibitory effect of 6G11 on FcγRIIB were analyzed via Western blot (Figure 5C). Only for the S2B‐W104A‐6G11 fusion, a change from receptor phosphorylation to signal inhibition was observed after MMP‐9 digestion.

## 3 Discussion

In this study, we established a strategy, exemplified by the therapeutic antibody 6G11 as a target for masking, to isolate an affinity‐based masking domain based on chicken scFvs obtained from a yeast immune library via a competition‐based screening approach. Due to the large evolutionary distance between humans and chickens, antibodies obtained from this immunization should cover a wide spectrum of epitopes compared to approaches utilizing mammals. The titer after the 4th booster immunization indicated a strong immune response against the 6G11 scFv. The observation could be confirmed by an enrichment of binding variants already after only one sorting round. This finding could be caused either by a generally strong immune response against the 6G11 scFv or the presence of only a few dominant, high affine variants. To investigate this question, next‐generation sequencing after library generation could be employed to give an insight into overall diversity.

By introducing the scFvs in the yeast surface display plasmid with the T2A/tGFP system, surface presentation during the screening process could be monitored via intracellular tGFP fluorescence [39]. This in turn enabled a staining procedure that included FcγRIIB in the screening process. The addition of FcγRIIB allowed the identification and isolation of variants interfering with the 6G11:FcγRIIB interaction. With this competitive setup, the identification of highly potent masking domains can be implemented into the screening procedure, which has not been reported in other approaches dealing with affinity‐based masking domains [5, 6, 7]. YSD is particularly suitable for this experimental setup due to its combination with flow cytometry, enabling the detection of several signals on the single‐cell level. The presented screening method might be generalized to facilitate lead candidate identification. In addition, this approach could be utilized for the identification and isolation of variants with different degrees of interference in the interaction between the antibody and antigen. For optimal masking and regain of function upon demasking, a balanced affinity, achieved in this work post‐screening through rational design, may be required that allows for sufficient off‐tumor masking but regain of binding in the TME.

In the case of our study, an scFv that does not interfere with the 6G11:FcγRIIB interaction but binds specifically to 6G11 and no other IgG antibodies was isolated to act as an anti‐idiotypic and functional negative control for the fusion constructs. Interestingly, nearly the whole population in the last screening round was able to efficiently block the 6G11:FcγRIIB interaction, indicating that variants binding outside of the 6G11:FcγRIIB interface seem to be underrepresented. This finding could be explained either by a few dominant variants in the early immune response as described above or high immunogenicity of the 6G11 CDRs. This finding importantly supports chicken immunization and subsequent YSD screening as a straightforward and expeditious route for the isolation of function‐blocking anti‐idiotype antibodies to a given monoclonal antibody.

Our screening results revealed that an anti‐idiotypic binding domain—in the sense that it specifically interacts with a particular monoclonal antibody—is not automatically able to prevent the binding of the mAb to its target. While the masking unit S2B obtained by library screening can efficiently block the 6G11:FcγRIIB interaction, another scFv (S2N) does not interfere with FcγRIIB binding. Interestingly, both scFvs displayed anti‐idiotypic binding behavior since no binding toward kappa and lambda light chains of unrelated antibodies such as rituximab, matuzumab, and hCP‐LCE was observed (Figure S6).

After fusion of the scFvs to the 6G11 LC and confirming MMP‐9 cleavability, binding of the constructs prior and post MMP‐9 digestion to FcγRIIB was tested in BLI experiments. The K_D_ calculated for 6G11 is comparable to values found in the literature, indicating a correct experimental setup. By analyzing the K_D_ of the S2N construct, an affinity of 8.3 nM was calculated corroborating the notion that the fused masking domain addresses an epitope on the 6G11 V_H_ or V_L_ domain that is specific for 6G11 but distinct from the FcγRIIB binding site. In the case of S2B‐6G11, no binding was observed prior to MMP‐9 digestion. This finding was expected since the scFv was able to efficiently block the interaction between 6G11 and FcγRIIB in the yeast surface display setting. However, FcγRIIB binding was not restored after MMP‐9 digestion which is likely due to retained association of the 6G11:S2B complex. That equilibrium binding occurs was shown by the fact that S2B can be removed to a significant extent by contacting the complex with immobilized 6G11 (Figure S12D).

After confirming the binding of S2N‐6G11 prior and post MMP‐9 digestion as well as S2B‐6G11 binding after MMP‐9 digestion and partial removal of the scFv, cell binding experiments on Raji cells were conducted. The EC_50_ of 6G11 was calculated to be 0.4 nM, which is comparable to values found in the literature. The EC_50_ determined for S2N‐6G11 prior and post MMP‐9 cleavage was 2 nM for both constructs. The 5‐fold reduction of binding is caused either by an inhibitory effect of the linker or the remaining amino acids after MMP‐9 digestion or by the S2N scFv still bound to 6G11. While the binding curves of 6G11 and S2N‐6G11 prior to MMP‐9 digestion align well, after MMP‐9 digestion, increased RFU values at higher concentrations were detected. This phenomenon, which was also observed with MMP‐9 cleaved S2B‐6G11, could be caused by MMP‐9 mediated modification of the cell surface resulting in a slightly increased accessibility of the antibody to FcγRIIB. In the case of S2B‐6G11 prior to MMP‐9 digestion, no EC_50_ value could be calculated since no binding was observed even at higher concentrations. A 2700‐fold restoration of binding could be achieved through MMP‐9 digestion when comparing the concentration at the highest binding signal for the untreated construct with the concentration of the cleaved sample at equal signal strength. Despite this, with an EC_50_ of 24 nM, cell binding of the digested S2B‐6G11 was still 65‐fold decreased compared to unmodified 6G11. After partial removal of the scFv, binding was further enhanced to only a 15‐fold difference.

As desired, S2N‐6G11 samples prevented FcγRIIB activation like unmodified 6G11 in the functional phosphorylation assay while S2B‐6G11 did not. In contrast to the on‐cell binding experiments, there was no partial restoration of receptor blocking for S2B‐6G11 after MMP‐9 digestion. Apparently, the residual interference of the cleaved‐off S2B scFv is sufficient to abolish receptor inhibition by 6G11. While 6G11 is still detectable during on‐cell binding despite the high affinity of the S2B scFv, it negates the minimum required engagement of 6G11 with FcγRIIB to prevent activation by rituximab Fc. However, partial removal of severed S2B scFv was sufficient to show conditional restoration by full receptor blocking by the surplus of unoccupied 6G11.

To overcome the retained inhibitory effect of S2B toward 6G11 after MMP‐9 cleavage, affinity attenuation was pursued based on AlphaFold 3 prediction and visualization of the S2B and 6G11 interface and subsequent identification of potential key amino acids in the scFv mask required for binding. In order to generate sequences with a broad range in affinity attenuation, also positions outside of CDR‐H3, which usually engages the most with the antigen [40], were considered for alanine substitution. To avoid bias by relying only on one single prediction, which might not be able to display all likely interactions at once, rational selection was assisted by summarizing the hydrogen bonds and salt bridges in the scFv:mAb interface of multiple AlphaFold 3 models. Based on this, amino acids in S2B CDR‐H2 and CDR‐L1 with predicted hydrogen bonds to the 6G11 CDR‐H3 were included in the attenuation approach, besides disruption of a salt bridge or a hydrophobic interaction of S2B CDR‐H3 with 6G11 CDR‐L1 or FR‐L2, respectively. In total, four positions were selected for one alanine substitution per S2B sequence which were expressed as solitary scFvs for initial characterization. Melting points of all mutants were in the range of S2B, indicating no detrimental effect on protein stability. Measuring binding to 6G11 via ELISA showed up to 3‐fold attenuation regarding EC_50_s for the mutant scFvs, except for the S164A variant. BLI experiments revealed a 6‐ and 10‐fold decrease in affinity for the S2B R101A and S2B W104A scFvs, respectively. The S2B Y52A variant displayed a 4‐fold decrease in binding, while the S164A mutation again retained a similar affinity as the original S2B. Since different degrees of attenuation were achieved, light chain fusions were generated and characterized. Melting points of the light chain variants prior and post MMP‐9 digestion were comparable to S2B‐6G11, indicating equal protein stability. In the case of aggregation behavior, most light chain fusions of the mutants displayed a weak tendency for a decrease in monomer content after MMP‐9 incubation. This phenomenon might be caused by scFvs which do not remain bound to 6G11 after cleavage, as intended, hence resulting in an additional elution signal. Further investigation of antibody blocking and demasking in BLI experiments demonstrated no binding of the soluble extracellular FcγRIIB domain to 6G11 fused with any of the S2B variants. Recovery of receptor binding was observed for MMP‐9 treated constructs except when utilizing the S2B S164A mask. This was further investigated in cell binding experiments. Surprisingly, despite no influence by the S164A exchange on the K_D_ of the solitary scFv, cleaving of the MMP‐9 linker to the serine mutant did not allow cell binding of 6G11 in a concentration‐dependent manner as previously observed for the original S2B‐6G11 construct. How this single point mutation with marginal changes to the affinity of the scFv resulted here in a different masking efficiency remains unclear. In the case of the Y52A exchange, low blocking efficiency at higher concentrations but full recovery after MMP‐9 digestion was observed. This significant loss in masking was not expected since the solitary S2B Y52A scFv displayed in BLI only a 4‐fold reduction in 6G11 binding compared to the original S2B. As with the S2B S164A scFv, the precise underlying mechanism responsible for this observation remains unknown. One explanation might be an epitope drift caused by substitution‐induced structural changes in the S2B CDRs beyond the disruption of the modeled interactions with the 6G11 paratope. For the arginine mutant, incapable of forming the S2B R101–6G11 D34 salt bridge, binding is absent prior to MMP‐9 incubation, with partial restoration of receptor binding on target cells afterward, despite similar EC_50_ to the unmodified 6G11 control. In case of S2B‐W104A‐6G11, the best combination of blocking and demasking efficiencies out of all four variants was obtained, with only weak cell binding before and full restoration after MMP‐9 cleavage, regarding maximum binding signals and EC_50_s. Importantly, this W104A mutation in the S2B sequence allowed conditional activation of 6G11 function in vitro by a tumor‐associated protease, as shown by Western blot analysis of the FcγRIIB phosphorylation state in target cells, without the need to artificially remove the cleaved‐off mask. For the other alanine substitution variants, previous observations of low binding recovery of MMP‐9 treated constructs in the on‐cell binding experiment as well as the degree of attenuation determined for the solitary scFvs carried over to the functional in vitro assay. However, BLI measurements of receptor binding to the cleaved scFv 6G11 fusions showing recovery were not predictive for the phosphorylation assay since similar K_D_s were obtained.

It remains to be elucidated by animal studies whether full regain of antibody potency also occurs in the tumor microenvironment. In general, it may be necessary to find an “affinity window” for the respective masking unit that allows for efficient functional blocking of the target therapeutic antibody when covalently linked to it while proteolytic release should result in a significant drop of affinity such that the blocking unit is released and diluted in the tumor tissue. To achieve this, if required, attenuation of the affinity of an anti‐idiotype functional blocking scFv can easily be performed by introducing single amino acid exchanges preferably in CDR‐H3, as shown in this work, which is in most cases mainly responsible for antigen binding [41, 42]. Likewise, scFv humanization via CDR grafting and screening of a yeast display library with variegated key residues for CDR orientation also provides an opportunity to obtain less or more potently blocking variants during the FACS screening process [43]. Moreover, for fine‐tuning the function of the masking unit, it could also be advantageous to combine proteolytic demasking with pH‐dependent binding. By His‐doping of the CDRs, pH‐dependency can be introduced [44, 45], resulting in a masking scFv that displays reduced target binding in the acidic tumor microenvironment, thus supporting the release of proteolytically cleaved‐off scFv.

In conclusion, we established a procedure for expeditious isolation of scFv‐based masking units and subsequent fine‐tuning of demasking for conditional antibody activation in the tumor microenvironment that might contribute to an improved safety profile by limiting expected on‐target off‐tumor toxicity.

## 4 Material and Methods

### 4.1 Plasmids and Yeast Strains

Plasmids and methods for library generation by homologous recombination as well as for expression of scFvs in *E. coli* and antibodies in Expi293F HEK cells (Gibco, Thermo Fisher Scientific: A14527) were previously discussed in detail [44, 46, 47, 48]. Sequences for the antibodies 6G11, matuzumab, rituximab, and hCP‐LCE were derived from patents [32, 34, 36] and a publication [35], respectively.

### 4.2 Chicken Immunization

Chicken immunization was performed as previously described by Davids Biotechnologie GmbH (Regensburg, Germany) [49]. Briefly, a chicken was immunized five times (days 1, 14, 28, 42, and 56) with 6G11 scFv. The serum titer was determined after the fourth immunization by ELISA. On day 63 the animal was sacrificed and subsequent isolation of spleen cells as well as RNA isolation was performed. All previously described steps were conducted by Davids Biotech GmbH. Experimental procedures and animal care were in accordance with EU animal welfare protection laws and regulations.

### 4.3 Library Generation and Sorting

The isolated RNA was transcribed into cDNA using SuperScript III Reverse Transcriptase (Invitrogen: 1808051) according to the manufacturer's protocol and as described elsewhere [47]. V_H_ and V_L_ encoding genes were amplified and fused together by PCR and transformed into *Saccharomyces cerevisiae* yeast cells for homologous recombination as described [46, 47]. To monitor scFv expression, the plasmid additionally carried the T2A tGFP sequence resulting in coexpression of the antibody fragment and tGFP [39].

Library sorting (FACS: influx v7 sorter BD) was performed for two rounds utilizing 1 µM of 6G11 antibody. The yeast cells were washed twice with 1 mL PBS‐B (0.1% bovine serum albumin) before incubating with 6G11 for 30 min at room temperature (RT). After two subsequent washing steps with 1 mL PBS‐B, surface bound 6G11 was detected with anti‐human Fc‐PE antibody (eBioscience, Thermo Fisher Scientific: 12‐4998‐82), incubated for 15 min on ice. In round three, the 6G11 concentration was decreased to 100 nM and combined with 1 µM of His‐tagged human FcγRIIB extracellular domain protein (ACRO Biosystems: CDB‐H5228) and incubated for 30 min at RT. After two subsequent washing steps with 1 mL PBS‐B, secondary anti‐human Fc‐PE antibody (eBioscience, Thermo Fisher Scientific: 12 4998 82) as well as anti‐his‐Alexa647 antibody (QIAGEN: 35370) were applied and incubated for 15 min on ice.

### 4.4 Reformatting, Expression, and Purification of scFvs and Light Chain Fusions

Reformatting, expression, and purification of scFvs were performed as previously described [48]. 6G11 scFv was produced in the same fashion.

For light chain fusions, the scFvs were amplified utilizing the primers Chicken VL GG up (5′‐ATATATGCTCTTCAAGTGCGCTGACTCAGCCGTCC‐3′) and Chicken VL MMP‐9 lo (5′‐ATATATGCTCTTCAGAAGCCCAGGGGCATGTGCACGCTACCGCCGCCACCGCTGCCACCACCGCCTAGGACGGTCAGGGTTGTCCC‐3′). The 6G11 light chain was amplified using 6G11 LC MMP‐9 up (5′‐ATATATGCTCTTCATTCCTGGGCCCCGGCGGTGGCGGCAGCGGCGGTGGCGGTAGCGGTGGTCAATCAGTCCTCACTCAACCGCCT‐3′) and pTT5 Lambda GG lo (5′‐ATATATGCTCTTCTCGCACTATTAGCTGCACTCGGTGG‐3′). Assembly of light chain fusions in pTT5 was carried out via golden gate utilizing the SapI (NEB: R0569) restriction enzyme, 75 ng pTT5 plasmid, and a 3‐fold molar excess of each insert. Plasmids with the correct sequence were utilized for the transfection of Expi293F HEK cells (Gibco, Thermo Fisher Scientific: A14527). Transfection and antibody purification via Protein A (1 mL HiTrap Protein A HP, Cytiva: 29048576) using an ÄKTA start chromatography system (Cytiva) was performed as previously described [44].

### 4.5 Affinity Determination of scFvs via Enzyme‐Linked Immunosorbent Assay

For affinity determination, wells of a MaxiSorp 96‐well plate (Thermo Fisher Scientific: 442404) were coated with 100 µL of 5 µg/mL 6G11 solution in carbonate coating buffer (8.4 g/L NaHCO_3_ and 3.56 g/L Na_2_CO_3_ pH 9.5) overnight at 4°C. Afterward, each well was washed three times with 200 µL of PBST (PBS + 0.1% Tween‐20) and blocked with 200 µL of blocking solution (PBS + 1% BSA) for 1 h at RT. After three washing steps, 100 µL of scFvs were added in different concentrations (ranging from 500 to 0.19 nM) to different wells and incubated for 1 h at RT. After three subsequent washing steps, each well was incubated with a 1:10,000 dilution of StrepTactin‐HRP (Bio‐Rad: 1610381) for 1 h at RT. After five subsequent washing steps, 100 µL of TMB One solution (Promega: G7431) was added to each well, incubated until color change, and the reaction was stopped with 100 µL of a 1% HCl solution. Afterward, the absorbance at 405 nm was measured in the CLARIOstar Plus plate reader.

### 4.6 Affinity Determination, Specificity Testing, and Epitope Binning via Biolayer Interferometry

Epitope binning and affinity determination were performed using an Octet Red96 BLI Detection system (Forte BIO) as previously described [49]. For affinity determination of 6G11 and light chain fusions, Protein A (ProA) Biosensor tips (Sartorius: 18–5010) were loaded with 6G11 or the respective light chain construct (5 µg/mL). Afterward, tips were transferred into wells containing different FcγRIIB concentrations ranging from 125 to 15.6 nM in serial 1:2 dilutions for a 60 s association step. Subsequently, the tips were moved to a kinetics buffer to measure the dissociation rate for 40 s.

For specificity testing, Protein A biosensors were loaded with different antibodies (5 µg/mL) and subsequently transferred to a well containing either S2B or S2N scFv at a concentration of 2 µM. For epitope binning, 6G11 (5 µg/mL) was loaded to a Protein A biosensor and transferred to a well containing 2 µM of S2B scFv for 240 s, followed by a subsequent transfer to a well containing 2 µM of S2N scFv for 240 s.

For affinity determination of solitary scFvs, Protein A tips were loaded with 6G11 (5 µg/mL) and transferred to wells containing 1:2 serial dilutions (500–7.8 nM) of original or mutated S2B scFv. Association and dissociation were measured for 180 s each.

### 4.7 Size Exclusion Chromatography

For analytical SEC using an HPLC 1260 Infinity chromatography system (Agilent Technologies), 40 µg of each antibody were applied to a TSKgel SuperSW3000 column (Tosoh Bioscience) at a flow rate of 0.35 mL/min with TN buffer (50 mM Tris, 150 mM NaCl pH 7.4) as eluent. Protein elution was detected at 280 nm.

### 4.8 Thermal Shift Assay

For thermal shift assays using a C1000 thermal cycler with a CFX96 optical module (Bio‐Rad), 2 µL of 1:10 diluted SYPRO Orange were mixed with 18 µL of respective antibody (concentration 0.5–1 mg/mL) and the Hard‐Shell 96‐well PCR plate was sealed by a Microseal PCR plate sealing film. Fluorescence was constantly measured, while the temperature gradient was set from 20°C to 98°C with 0.5°C temperature increments per 30 s.

### 4.9 On‐Cell EC_50_ Determination

Measurement of on‐cell EC_50_ was performed as previously described [50]. Briefly, 250.000 Raji cells (FcγRIIB^+^ and CD20^+^) were transferred into wells of a 96‐well plate, washed once with 150 µL PBS‐B and incubated with different concentrations of 6G11 and light chain constructs (Concentration range: 1000–0.005 nM in 1:5 serial dilution). The cells were incubated for 30 min at RT and subsequently washed twice with 200 µL PBS‐B. Afterward, anti‐human Fc PE secondary antibody from goat was added to each well and incubation was carried out for 15 min on ice. After two additional washing steps, the cells were resuspended in 100 µL PBS‐B and analyzed via flow cytometry using a CytoFLEX S (Beckman Coulter).

### 4.10 Affinity Chromatography to Remove Cleaved‐Off scFv Mask

To remove the scFv mask from 6G11 samples after MMP‐9 cleavage, an affinity column was prepared by covalently binding 6G11 to 1 mL of NHS‐activated resin (Cytiva: 17071601). Digested samples were applied via a syringe and incubated for 10 min on the column at RT. The flow‐through was collected and applied again after washing the column with 3 mL PBS to dilute bound scFv. Samples were concentrated via spin concentrators (Amicon Ultra‐0.5 mL Centrifugal Filters 3 kDa MWCO, Sigma–Aldrich: UFC500308) and removal of scFv was estimated after SDS‐PAGE and Coomassie staining.

### 4.11 Determination of FcγRIIB Phosphorylation Status

FcγRIIB and CD20 positive Raji cells were cultivated in RPMI medium 1640 (Gibco, Thermo Fisher Scientific: 11875093) with 10% FBS, 1% Pen/Strep under standard conditions (37°C, 5% CO_2_ and humidified atmosphere). Per sample, 3•10^6^ viable cells were harvested (500 × *g*, 4 min at RT) and resuspended in 40 µL cultivation media. 0.5 µg of each antibody was prepared in 10 µL RPMI 10% FBS, 1% Pen/Strep in pre‐heated microcentrifuge tubes and mixed with 40 µL Raji cells to a final concentration of 10 µg/mL per antibody. Samples were incubated for 30 min at 37°C and 400 rpm (∼15 × *g*). The medium was removed after centrifugation at 800 × *g*, 2 min and the pellet was resuspended with 35 µL lysis mix containing PhosSTOP (Roche, Sigma–Aldrich: 4906845001) and Halt Protease and Phosphatase Inhibitor Cocktail (Thermo Fisher Scientific: 1861281) in tris lysis buffer (Meso Scale Diagnostics: R60TX‐3). After 10 min incubation on ice, samples were stored overnight (OVN) at ‐80°C. For polyacrylamide gel electrophoresis, samples were thawed at 37°C, and cell debris was pelleted at 13,000 rpm (∼17,000 × *g*) for 2 min. A total of 12.5 µL lysate was mixed with 3 µL of 5× reducing Laemmli loading buffer and denaturated at 98°C for 10 min. SDS‐PAGE (15% resolving gel) was run at 300 V and 45 mA for 30–45 min with the Amersham Biosciences system. The acrylamide gel and a nitrocellulose membrane (Amersham Protran 0.45 µm NC, Cytiva: 10600002) were incubated for 1 min in transfer buffer (25 mM Tris, 192 mM glycine, 20% methanol, pH 8). Western blotting was performed at 25 V and 300 mA for 35 min with the Bio‐Rad Trans‐Blot Turbo system. The membrane was washed three times with TBST (tris buffered saline, 0.1% Tween 20, pH 8) and blocked OVN in 10 mL TBST‐B with 5% bovine serum albumin (BSA) at 4°C (60 rpm, 50 mL tube rotator). Staining was performed in two steps for 1 h each at RT with tree 1 min washing steps (TBST) in between. For the first staining, 10 mL of 1:100.000 dilution (9 ng/mL) of anti‐CD32B (phospho Y292) antibody (rabbit, Abcam: ab68423, RRID:AB_1209223) and 1 : 50,000 (20 ng/mL) anti‐alpha‐tubulin antibody (rabbit, Sigma–Aldrich: SAB3501072) were applied in TBST‐B. The second staining used 5 mL TBST‐B with 1:5000 anti‐rabbit IgG, AP‐linked antibody (goat, Cell Signaling Technology: 7054). For signal generation, 5 mL of 1‐Step NBT/BCIP‐substrate solution (Thermo Fisher Scientific: 34042) were applied for 10–20 min. The reaction was stopped with water and the membrane was scanned for analysis via ImageJ. For analysis of biological replicates (*n* = 3), one‐way ANOVA (GraphPad Prism v10).

### 4.12 Simulation and Analysis of the S2B scFv:6G11 Interface

AlphaFold Server (using AlphaFold 3 [37]) was used for structure prediction of the anti‐idiotypic binding of S2B scFv to 6G11. As input, the S2B scFv, the 6G11 V_H_‐C_H_1, and the 6G11‐LC were submitted as separate sequences without the full‐length MMP‐9 cleavable linker or its fragments. The resulting 25 structure predictions from five modeling jobs were superimposed and analyzed in UCSF ChimeraX version 1.7 [51]. Per model, only hydrogen bonds and salt bridges of the scFv with the 6G11 chains were predicted (command prompt: hb/a intermodel false restrict cross) and exported for all structures in summary. In this data set, the number of sidechain‐sidechain interactions per S2B scFv amino acid position was calculated.

## Author Contributions

Jan Habermann: Conceptualization (equal); investigation (equal); visualization (equal); writing—original draft (equal). Dominic Happel: Conceptualization (equal); investigation (equal); visualization (equal); writing—original draft (equal). Adrian Bloch: Investigation (supporting); writing—review and editing (supporting). Charles Shin: Conceptualization (supporting). Harald Kolmar: Conceptualization (equal); project administration (lead); writing—original draft (equal).

## Ethics Statement

Handling, immunization, and sacrifice of one chicken was performed by Davids Biotechnologie GmbH (Regensburg, Germany). Experimental procedures and animal care were in accordance with EU animal welfare protection laws and regulations. Ethical approval waived due to national legislation of Lower Franconia (Germany); reference: AZ 55.2.2‐2532.2‐859‐21.

## Conflicts of Interest

The authors declare that the research was conducted in the absence of any commercial or financial relationships that could be construed as a potential conflict of interest.

## Supporting information



Supporting Information

## Data Availability

The data that supports the findings of this study are available in the supplementary material of this article.
