# Supplementary material for: A Competition‐Based Strategy for the Isolation of an Anti‐Idiotypic Blocking Module and Fine‐Tuning for Conditional Activation of a Therapeutic Antibody
Source: Biotechnol J. 2024 Dec 10;19(12):e202400432. doi: 10.1002/biot.202400432 (PMC11629141; doi:10.1002/biot.202400432)
Supplement: Supplementary file 1 — Supporting Information [file BIOT-19-e202400432-s001.pdf]

Supporting Information

A competition-based strategy for the isolation of an anti-idiotypic blocking module and fine-tuning for conditional activation of a therapeutic antibody

Jan Habermann[1†], Dominic Happel[1†], Adrian Bloch[1], Charles Shin[2], Harald Kolmar[1,3,*]

[1]Institute for Organic Chemistry and Biochemistry, Technical University of Darmstadt, Darmstadt, Germany

[2]Department of Biomedical Engineering, Johns Hopkins University, Baltimore, MD, USA

[3]Centre for Synthetic Biology, Technical University of Darmstadt, Darmstadt, Germany

†These authors contributed equally to this work and share first authorship

***Correspondence:**

Harald Kolmar, Institute of Organic Chemistry and Biochemistry, Technical University of Darmstadt, Peter-Gruenberg-Str. 4, D-64289 Darmstadt.

Email: Harald.Kolmar@tu-darmstadt.de

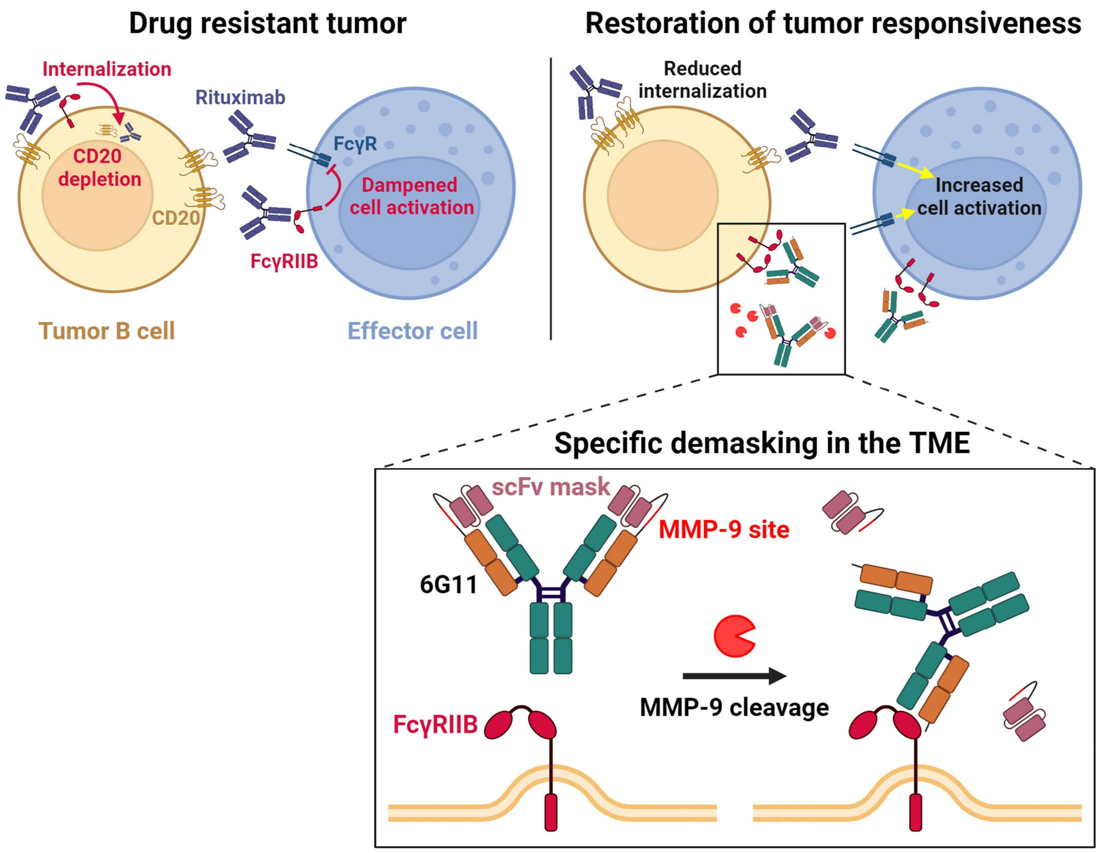

Figure S1: Schematic depiction of a masked variant of the therapeutic antibody 6G11 to restrict FcγRIIB binding to the tumor site. (Top left) B cell lymphoma can develop a resistance to therapeutic antibodies like rituximab (violet) by FcγRIIB (dark red) mediated internalization of the drug and its target, here CD20 shown in orange. Additionally, overexpression of this inhibitory Fcγ receptor in the tumor microenvironment down-regulates anti-tumor responses dependent on activatory Fc receptors (blue). (Top right) Blocking of FcγRIIB with the antibody 6G11 negates these tumor escape mechanisms [1, 2]. (Zoomed in inset) To spare healthy cells, 6G11 was extended in this work with an anti-idiotypic single chain Fv (scFv) to block the paratope while a linker with a cleavage site for the tumor microenvironment associated protease MMP-9 allows demasking at the tumor site [3]. Concept created with BioRender.

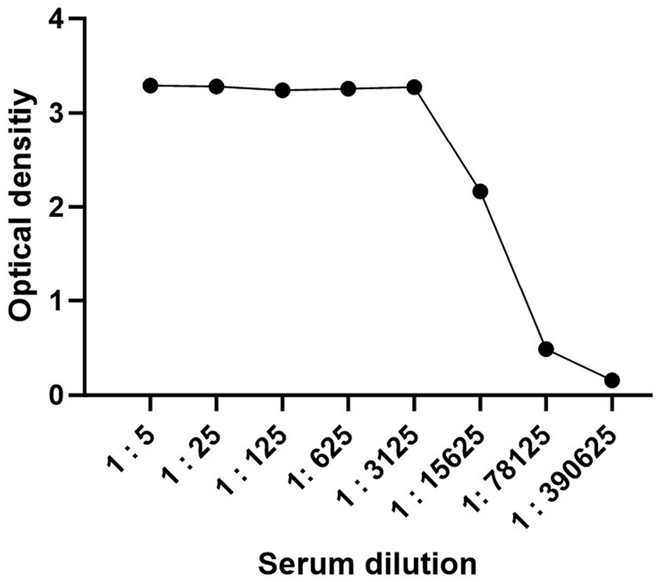

Figure S2: ELISA-based titer determination of the immunized chicken. Chicken immunization and ELISA performed by Davids Biotech. A serum sample was collected after the third booster with 6G11 scFv. Binding towards 6G11 was assessed by an ELISA based assay utilizing a 1:5 serial serum dilution.

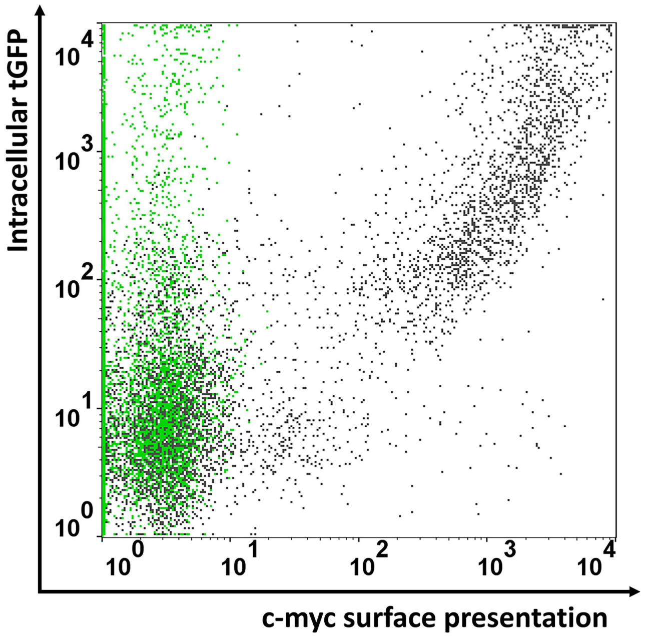

Figure S3: Flow cytometry analysis of correlation between c-myc surface presentation and intracellular tGFP expression. Cells without staining are depicted in green, while the sample incubated with anti c-myc biotin and streptavidin allophycocyanin is shown in black.

Clone	Sequence
S2B VH	ASAVTLDESGGGLQTPGGGLSLVCKASGFTFSSYSMVWVRQAPSKRLEWVAGIYSSATWTYYAPAVKGRATISRDNGQSTVRLQLNDLRAEDTGTYFCAKTSRSGWTAYSASAIDAWGHGTEVIV
S2N VH	ASAVTLDESGGGLQTPGGGLSLVCKASGFSFSDRGMQWVRQAPGKGLEWVAGIDDDDNNTWYATAVKGRATISRDNGQSTVRLQLNSLRAEDTGTYYCAKTPTSYY--WGAAEIDAWGHGTEVIV

Clone	Sequence
S2B VL	ALTQPSSVSANPGETVKITCSGGSSYYGWYQQKSPGSAPVTVIYSNDKRPSDIPSRFSGSTSGSTATLTITGVQADDEAVYFCGGYDSNIHGGIFGAGTTLTVL
S2N VL	ALTQPSSVSANPGETVKITCSGSSGSYGWYQQKAPGSAPVTVIYDNNNRPSNIPSRFSGSLSGSTNTLTITGVQADDEAVYFCGSYEGSSYVGIFGAGTTLTVL

Figure S4: Single letter amino acid alignment of the V_H and V_L domain of S2B and S2N scFvs. Framework regions are depicted in black, while CDRs identified according to the Kabat and IMGT numbering scheme are shown in red and underlined, respectively.

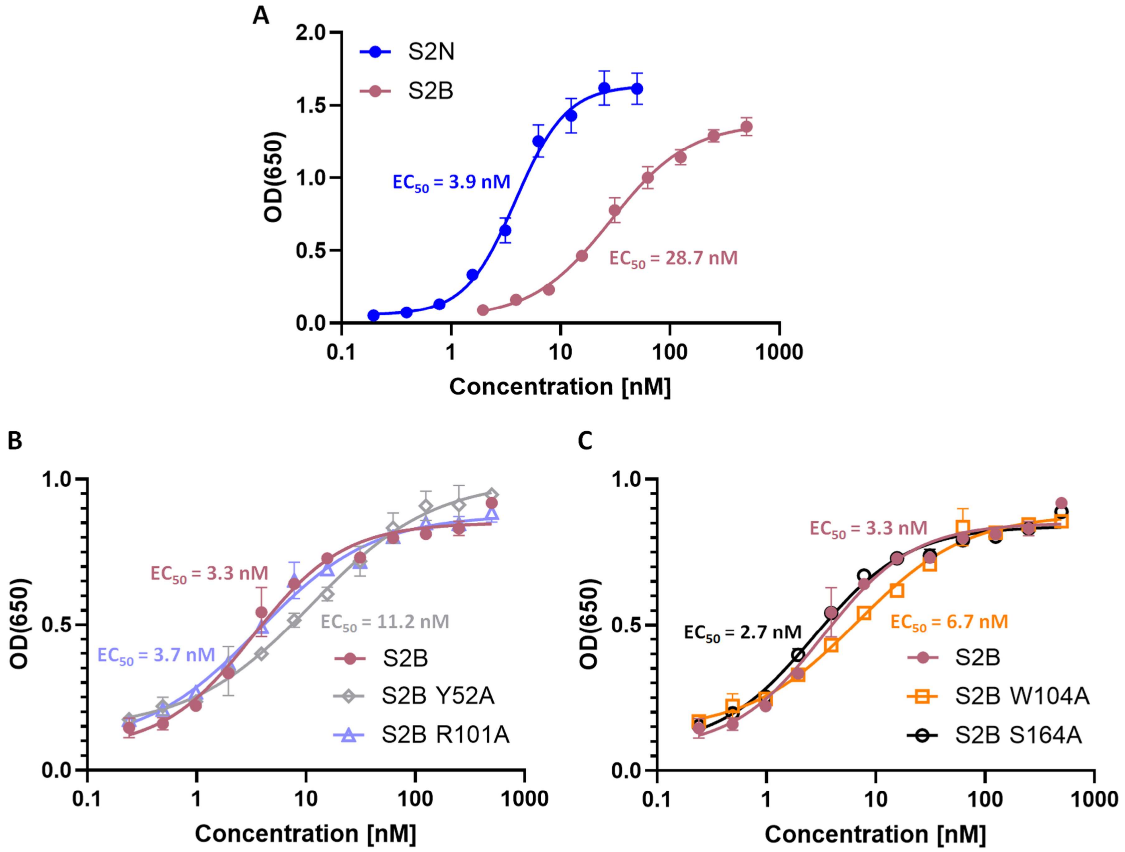

Figure S5: EC_{50} determination of the scFvs S2N, S2B, and S2B mutant *via* ELISA. The logarithmic concentration is shown on the x-axis, whereas the optical density at 650 nm is depicted on the y-axis. (A) The binding curves for S2B and S2N are represented in mauve and blue, respectively. (B) S2B, S2B Y52A, and S2B R101A binding curves are depicted in mauve, light grey, and pale blue, respectively. (C) The binding curves for S2B, S2B W104A, and S2B S164A are illustrated in mauve, orange, and black, respectively.

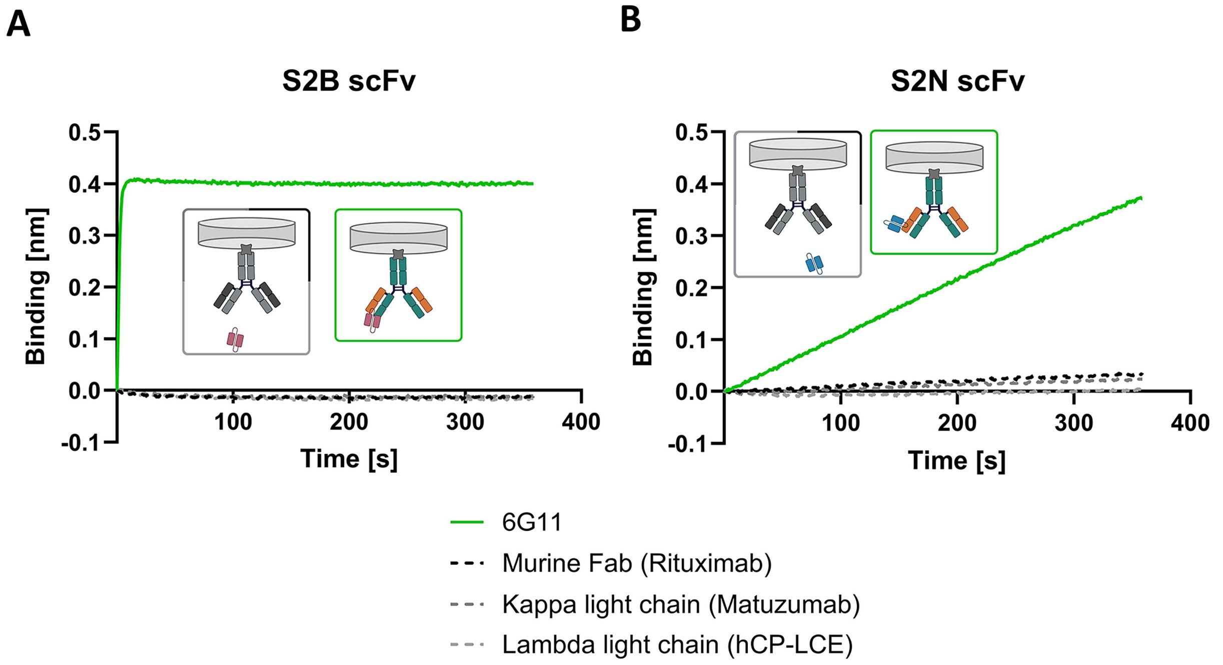

Figure S6: Testing the binding specificity of isolated scFvs S2B and S2N in BLI experiments in the presence of different antibodies. The binding signal in nm is displayed on the y-axis, while the time in seconds is shown on the x-axis. The respective antibody was loaded to the biosensor with a subsequent association step of the scFv. (A) Binding of the S2B scFv to different antibodies. Binding of 6G11 is shown in green, while binding to rituximab, matuzumab and hCP-LCE are depicted in black, dark grey and light grey respectively. (B) Binding of the S2N scFv to different antibodies. Binding of 6G11 is shown in green, while binding of rituximab, matuzumab and hCP-LCE are depicted in black, dark grey and light grey, respectively.

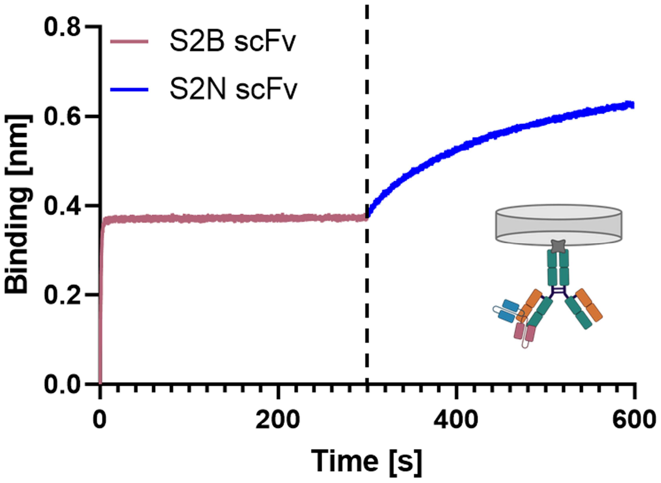

Figure S7: Investigating binding properties of S2B and S2N scFvs in BLI experiments. The binding signal in nm is displayed on the y-axis, while the time in seconds is shown in the x-axis. The transition between the different steps is marked by the dashed line. 6G11 is loaded to a biosensor, binding of S2B (2 µM) is depicted in mauve, while subsequent S2N binding (2 µM) is shown in blue.

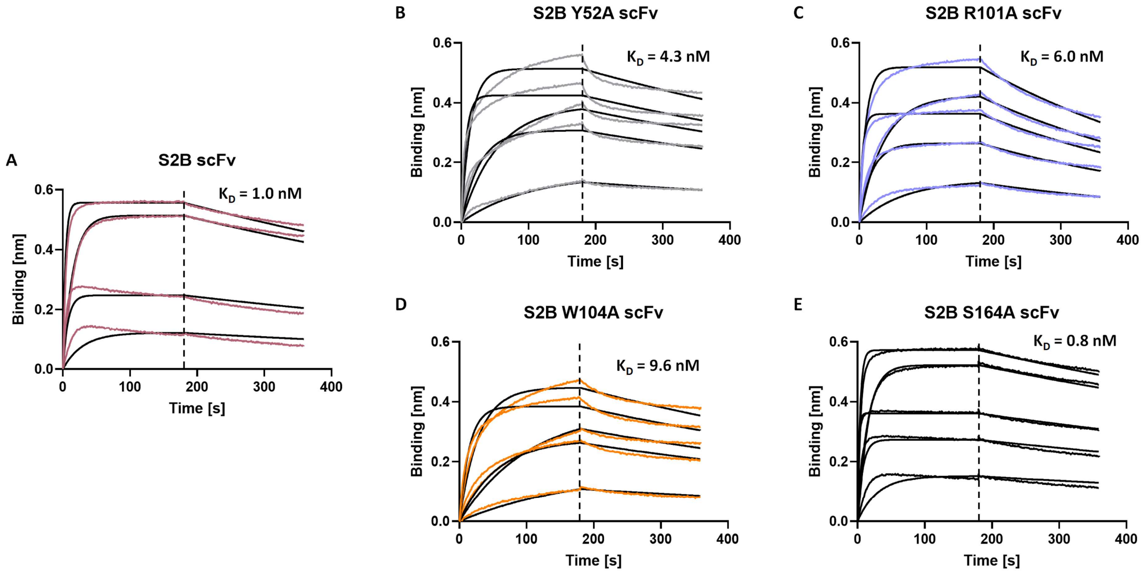

Figure S8: Binding kinetics of S2B scFv and its mutants measured *via* BLI. The y-axis represents binding in nanometers, while the x-axis represents time in seconds. 6G11 is loaded to the BLI tip and followed by scFv incubation in different concentrations ranging from 500 nM to 31.3 nM. Fits for affinity determination are shown in black. (A) Binding kinetic of the S2B scFv is shown in mauve. (B) Binding kinetic of the S2B Y52A mutant is depicted in light grey. (C) Binding kinetic of the S2B R101A mutant is shown in light purple. (D) Binding kinetic of the S2B W104A mutant is depicted in orange. (E) Binding kinetics of the S2B S164A mutant displayed in black.

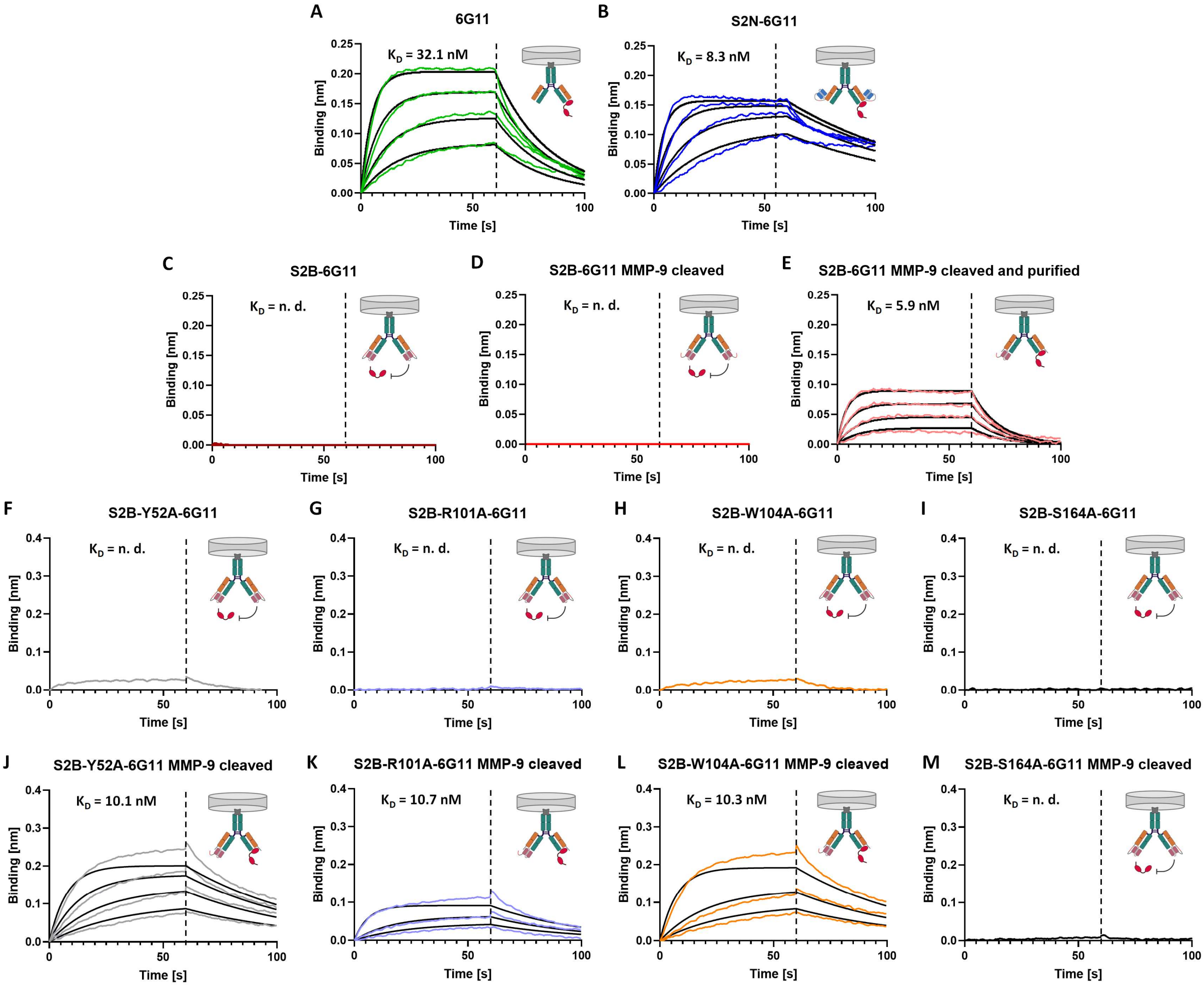

Figure S9: Binding kinetics of 6G11 light chain fusions measured *via* BLI. The y-axis represents binding in nanometers, while the x-axis represents time in seconds. 6G11 or light chain fusions are loaded to the BLI tip followed by FcγRIIB incubation in a concentration range from 500 nM to 31.3 nM. Fits for affinity determination are shown in black. (A) Binding kinetic of 6G11 is depicted in green. (B) Binding kinetic of the S2N-6G11 light chain fusion is displayed in blue. (C) Binding kinetic of the S2B-6G11 light chain fusion is shown in dark red. (D) Binding kinetic of the S2B-6G11 light chain fusion after MMP-9 digestion is displayed in red. (E) Binding kinetics of the S2B-6G11 light chain fusion after MMP-9 digestion and partial removal of the scFv is shown in light red. (F–I) Binding kinetics of the S2B-6G11 mutant light chain fusions are shown in grey, light purple, orange, and black, respectively. (J–M) Binding kinetics of the S2B-6G11 mutant light chain fusions after MMP-9 digestion are displayed in grey, light purple, orange, and black, respectively.

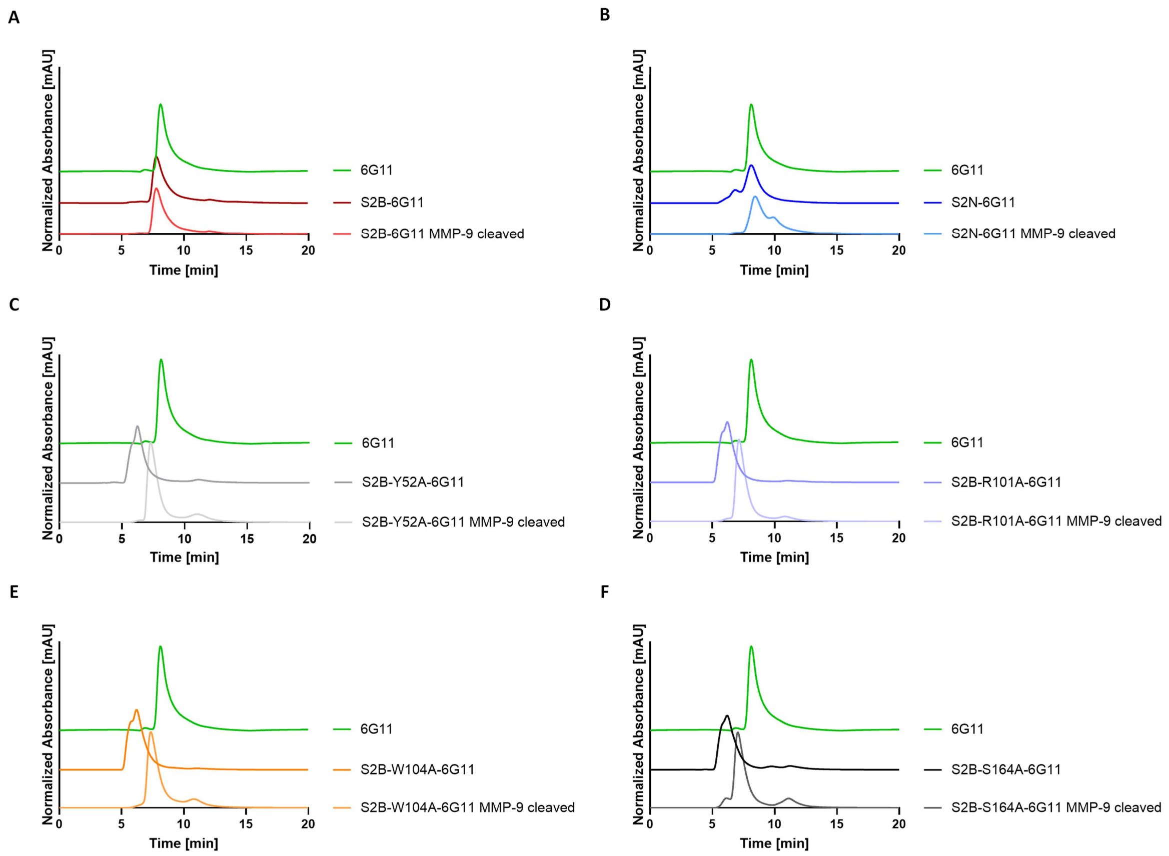

Figure S10: Analysis of 6G11 light chain fusions by analytical size exclusion chromatography (SEC). The normalized absorbance in mAU is shown in the y-axis, while the time in seconds is displayed on the x-axis. 6G11 (green) is shown in each graph as a reference. Light chain fusions prior and post MMP-9 cleavage are displayed by darker and the corresponding lighter color, respectively. (A) S2B-6G11 light chain fusion. (B) S2N-6G11 light chain fusion. (C) S2B-Y52A-6G11 light chain fusion. (D) S2B-R101A-6G11 light chain fusion. (E) S2B-W104A-6G11 light chain fusion. (F) S2B-S164A-6G11 light chain fusion.

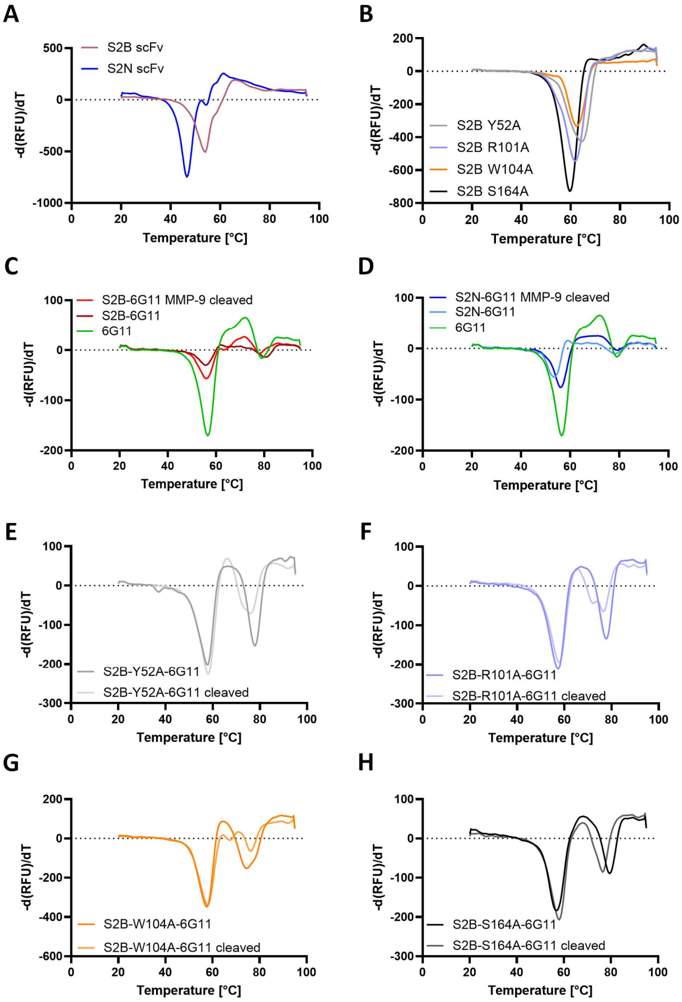

Figure S11: Melting point analysis of 6G11 light chain fusions and anti-idiotypic scFvs. The y-axis of the graphs displays the derivative of fluorescence decrease, while the x-axis shows the temperature. Light chain fusions prior and post MMP-9 cleavage are displayed by darker and the corresponding lighter color, respectively. (A) Melting point analysis of S2B and S2N. scFvs are shown in mauve and blue, respectively. (B) Analysis of the thermal stability of S2B mutants. S2B Y52A, S2B R101A, S2B W104A, and S2B S164A are displayed in grey, light purple, orange, and black, respectively. (C) S2B-6G11 light chain fusion with 6G11 as a reference shown in green. (D) S2N-6G11 light chain fusion with 6G11 as a reference shown in green. (E) S2B-Y52A-6G11 light chain fusion. (F) S2B-R101A-6G11 light chain fusion. (G) S2B-W104A-6G11 light chain fusion. (H) S2B-S164A-6G11 light chain fusion.

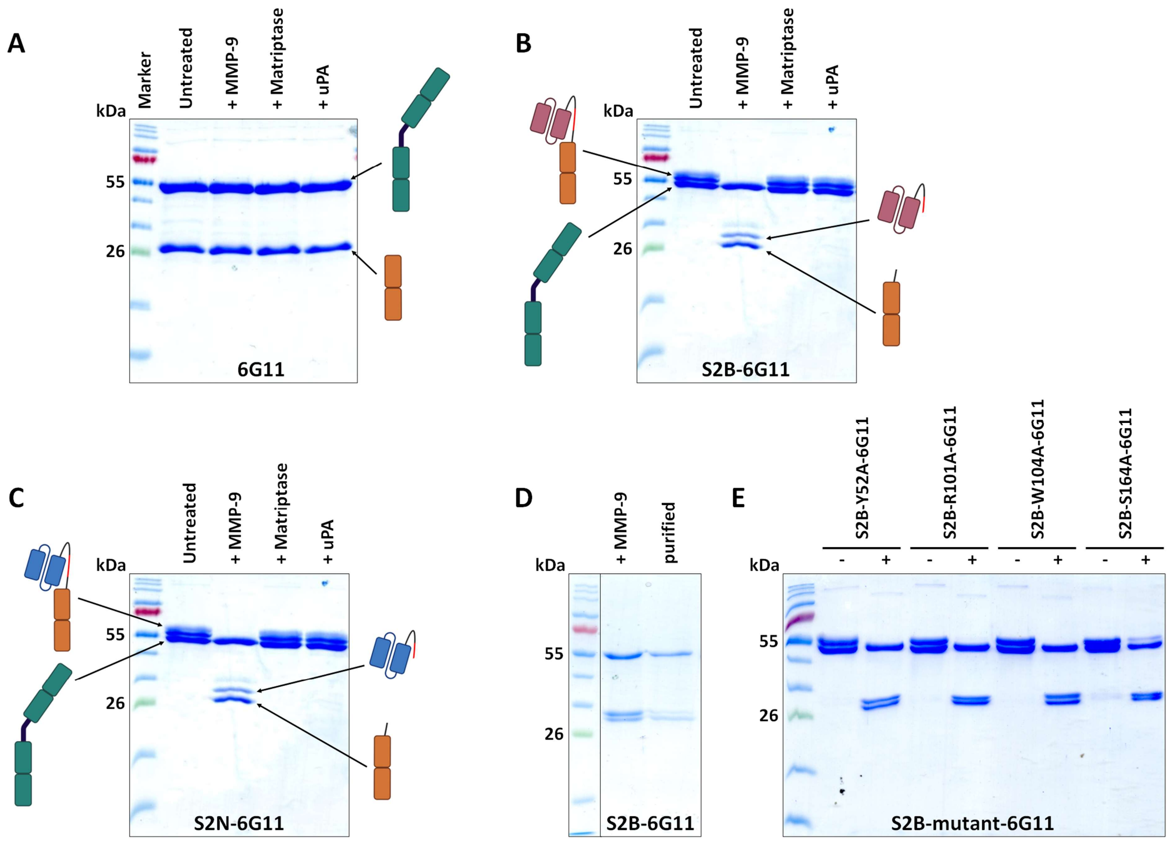

Figure S12: Exploration of protease stability of the 6G11 light chain fusions. S2B- and S2N-6G11 light chain constructs, as well as unmodified 6G11 (expected size heavy chain: 49.4 kDa, light chain: 23 kDa) were analyzed for scFv and linker stability utilizing either matriptase, urokinase-type plasminogen activator (uPA) or MMP-9. (A) Investigation of 6G11 upon protease treatment. (B) Analysis of S2B light chain fusion (lane 2–5, expected size heavy chain: 49.4 kDa, light chain linker: 50.5 kDa, MMP-9 cleavage mixture: light chain: 24 kDa, scFv fragment: 26.6 kDa) incubated with different proteases. (C) S2N light chain fusions containing the MMP-9 cleavable linker (lane 2–5, expected size heavy chain: 49.4 kDa, light chain linker: 50.3 kDa, MMP-9 cleavage mixture: light chain: 24 kDa, scFv fragment: 26.4 kDa) incubated with different proteases. (D) S2B light chain fusion after MMP-9 digestion and removal of the scFv *via* an 6G11-based affinity column. (E) Analysis of S2B mutants fused to the light chain of 6G11 prior and post MMP-9 cleavage.

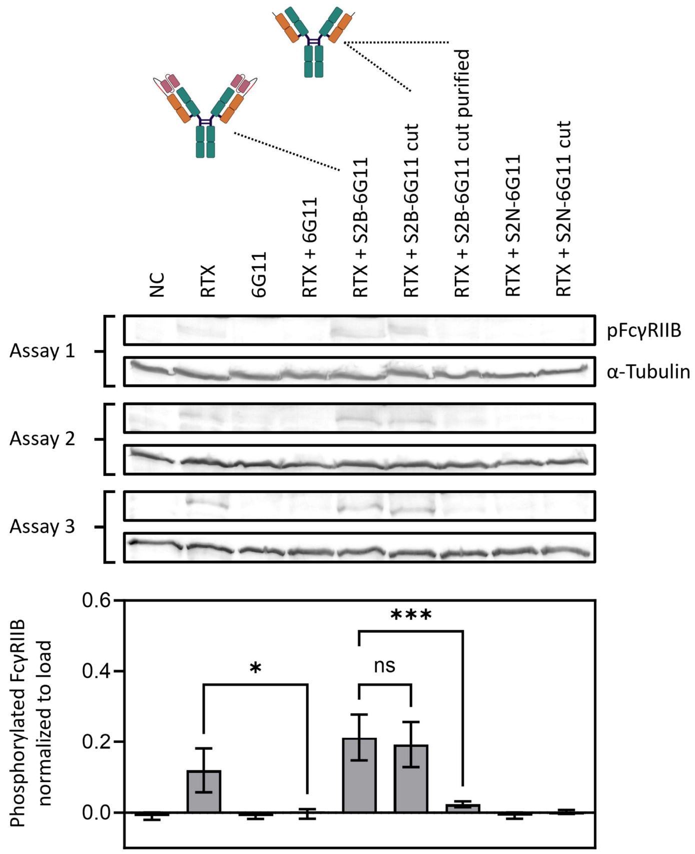

Figure S13: Ability of paratope blocking scFv S2B to prevent 6G11 binding to FcγRIIB on target cells. Western Blot analysis of phosphorylation state of FcγRIIB in presence of rituximab (RTX) alone and in combination with Fc silenced 6G11 variants on Raji cells. S2B-6G11: anti-idiotypic and blocking S2B scFv LC fusion. S2N-6G11: anti-idiotypic but non-blocking S2N scFv LC fusion as control. cut: unpurified MMP-9 digestion mix. cut + purified: scFv partial removed *via* 6G11-affinity column prior to loading. Staining with anti-FcγRIIB (phospho Y292) antibody from rabbit and anti-rabbit-IgG-AP. Normalization of phosphorylation signal to α-Tubulin loading control *via* ImageJ. Mean + SD of three independent experiments, depicted as Assay 1, Assay 2, and Assay 3. Statistical analysis *via* one-way ANOVA (Significance: $^*p \leq 0.05$, $^{**}p \leq 0.01$, $^{***}p \leq 0.001$).

Supplementary Table 1: Characterization of anti-6G11 chicken scFvs and light chain fusions. The table summarizes key metrics, including EC_{50} values for the light chain fusions obtained from cell binding assays, affinities determined by biolayer interferometry (BLI), melting temperatures measured *via* thermal shift assays, and aggregation levels assessed by size exclusion chromatography (SEC). For the scFvs, EC_{50} values were measured using ELISA, affinities were calculated from BLI data, and thermal stability was determined through thermal shift assays.

**Construct/Parameter**	**EC_{50} [nM]**	**K_D [nM]**	**Melting temp. [°C]**	**Aggregates [%]**
S2B scFv	3.3/28.7	1.0	59.0	–
S2B Y52A scFv	11.2	4.3	64.5	–
S2B R101A scFv	3.7	6.0	62	–
S2B W104A scFv	6.7	9.6	62.5	–
S2B S164A	2.7	0.8	59.5	–
S2N scFv	3.9	–	51.5	–
6G11	0.4	32.1	56.5	2.4
S2B-6G11	n. d.	n. d.	55.5	1.4
S2B-6G11 MMP-9 cleaved	26.0	n. d.	56.0	3.3
S2B-6G11 MMP-9 cleaved purified	6.0	5.9	–	–
S2B-Y52A-6G11	143.4	n. d.	57.5; 78	3.5
S2B-Y52A-6G11 MMP-9 cleaved	1.4	10.1	58; 76	12
S2B-R101A-6G11	n. d.	n. d.	57.5; 77.5	1.7
S2B-R101A-6G11 MMP-9 cleaved	1.0	10.7	58; 76.5	3.4
S2B-W104A-6G11	n. d.	n. d.	57.5; 74.5	0
S2B-W104A-6G11 MMP-9 cleaved	0.8	10.3	58; 76	12
S2B-S1S4A-6G11	n. d.	n. d.	57; 79.5	4
S2B-S1S4A-6G11 MMP-9 cleaved	n. d.	n. d.	58; 76.5	16.5
S2N-6G11	2.0	8.3	53.5	18.2
S2N-6G11 MMP-9 cleaved	2.0	n. d.	56.0	32.1

References

[1] Roghanian, A., Teige, I., Mårtensson, L., Cox, K. L. *et al.*, Antagonistic Human FcγRIIB (CD32B) Antibodies Have Anti-Tumor Activity and Overcome Resistance to Antibody Therapy In Vivo. *Cancer Cell* 2015, *27*, 473–488, DOI: 10.1016/j.ccell.2015.03.005.

[2] Karlsson, I., Gertsson, S., Kilany, S., Borggren, M. *et al.*, Phase 1/2a Clinical Trial of BI-1206, a Monoclonal Antibody to Fcγriib, in Combination with Rituximab in Subjects with Indolent B-Cell Non-Hodgkin Lymphoma That Has Relapsed or Is Refractory to Rituximab. *Blood* 2021, *138*, 1354, DOI: 10.1182/blood-2021-147452.

[3] Geiger, M., Stubenrauch, K.-G., Sam, J., Richter, W. F. *et al.*, Protease-activation using anti-idiotypic masks enables tumor specificity of a folate receptor 1-T cell bispecific antibody. *Nature communications* 2020, *11*, 3196, DOI: 10.1038/s41467-020-16838-w.
